# Barriers and enablers for deprescribing benzodiazepine receptor agonists in older adults: a systematic review of qualitative and quantitative studies using the theoretical domains framework

**DOI:** 10.1186/s13012-022-01206-7

**Published:** 2022-07-08

**Authors:** Perrine Evrard, Catherine Pétein, Jean-Baptiste Beuscart, Anne Spinewine

**Affiliations:** 1 Clinical Pharmacy Research Group, Louvain Drug Research Institute, Université catholique de Louvain, Brussels, Belgium; 2 Univ. Lille, CHU Lille, ULR 2694 - METRICS: Évaluation des technologies de santé et des pratiques médicales, F-59000 Lille, France; 3 Pharmacy Department, CHU UCL Namur, Yvoir, Belgium

**Keywords:** Benzodiazepines, Deprescribing, Older adults, Barriers and enablers to implementation, Theoretical domains framework

## Abstract

**Background:**

Many strategies aimed at deprescribing benzodiazepine receptor agonists (BZRA) in older adults have already been evaluated with various success rates. There is so far no consensus on which strategy components increase deprescribing the most. Yet, despite an unfavourable benefit-to-risk ratio, BZRA use among older adults remains high. We systematically reviewed barriers and enablers for BZRA deprescribing in older adults.

**Methods:**

Two reviewers independently screened records identified from five electronic databases—Medline, Embase, PsycINFO, CINAHL and the Cochrane library—and published before October 2020. They searched for grey literature using Google Scholar. Qualitative and quantitative records reporting data on the attitudes of older adults, caregivers and healthcare providers towards BZRA deprescribing were included. Populations at the end of life or with specific psychiatric illness, except for dementia, were excluded. The two reviewers independently assessed the quality of the included studies using the mixed-methods appraisal tool. Barriers and enablers were identified and then coded into domains of the theoretical domains framework (TDF) using a combination of deductive and inductive qualitative analysis. The most relevant TDF domains for BZRA deprescribing were then identified.

**Results:**

Twenty-three studies were included 13 quantitative, 8 qualitative and 2 mixed-method studies. The points of view of older adults, general practitioners and nurses were reported in 19, 9 and 3 records, respectively. We identified barriers and enablers in the majority of TDF domains and in two additional themes: “patient characteristics” and “BZRA prescribing patterns”. Overall, the most relevant TDF domains were “beliefs about capabilities”, “beliefs about consequences”, “environmental context and resources”, “intention”, “goals”, “social influences”, “memory, attention and decision processes”. Perceived barriers and enablers within domains differed across settings and across stakeholders.

**Conclusion:**

The relevant TDF domains we identified can now be linked to behavioural change techniques to help in the design of future strategies and health policies. Future studies should also assess barriers and enablers perceived by under-evaluated stakeholders (such as pharmacists, psychiatrists and health care professionals in the hospital setting).

**Trial registration:**

This work was registered on PROSPERO under the title “Barriers and enablers to benzodiazepine receptor agonists deprescribing”. Registration number: CRD42020213035

**Supplementary Information:**

The online version contains supplementary material available at 10.1186/s13012-022-01206-7.

## Contribution to the literature

- This systematic review identifies and synthesises barriers and enablers of BZRA deprescribing in older adults and map them into the theoretical domains framework.
- By including both qualitative and quantitative studies, this systematic review synthesises a variety of points of view and provides a deeper understanding of BZRA deprescribing implementation challenges.
- The identification of theoretical domains framework relevant domains can now be used for the theoretically informed development of future strategies towards BZRA deprescribing in older adults.

## Background

Benzodiazepine receptor agonists (BZRA, namely benzodiazepines and Z-drugs such as zolpidem, zopiclone and zaleplon) are widely prescribed for the management of insomnia and anxiety. However, their benefit-to-risk ratio is unfavourable in older adults (aged 65 or older) [1]. Indeed, BZRA offer only modest, short-term benefits, and adverse effects can include over-sedation, dependence, increased risks of falls and fractures, and cognitive impairment [2–4]. In Europe and the USA, recent studies have reported that around one in five adults aged 65 and older use BZRA [5–8]. Moreover, the proportion of long-term users among older individuals is estimated to be 47% [9]. In the nursing home (NH) setting, the prevalence of use is likely even higher, with reported rates between 14.6 and 54.4% [10–13]. For all these reasons, the American Geriatrics Society has included BZRA on the potentially inappropriate medications list [14] and the Screening Tool of Older Persons’ Prescriptions (STOPP) list version 2 recommends limiting their use to 4 weeks [15]. Moreover, several organisations such as the Canadian deprescribing network or Choosing Wisely recommend that deprescribing be offered to older adults who take BZRA [16, 17].

Deprescribing is the process of “withdrawal of an inappropriate medication, supervised by a healthcare professional with the goal of managing polypharmacy and improving outcomes” [18]. Many approaches to enhance implementation of BZRA deprescribing have already been evaluated, encompassing medication review, educational programmes, substitution or multi-faceted strategies [19–22]. These strategies were associated with discontinuation rates ranging from 27 to 80% [21]. However, routine implementation of such deprescribing strategies is limited, and deprescribing policies vary across countries [23]. Moreover, data remain limited on how to best achieve BZRA deprescription or which components of a strategy are the most effective.

Improving knowledge about the barriers and enablers of BZRA deprescribing could enhance the probability of success of strategies. Different stakeholders can be involved in BZRA deprescribing (e.g., the patient, relatives and informal caregivers, general practitioner (GP), nurses, pharmacist). Each of these stakeholders may perceive different barriers and enablers, which should all be assessed and taken into consideration [24]. A recent systematic review evaluated barriers and enablers for BZRA deprescribing in older adults [25]. Based on 10 included studies, the authors reported barriers such as the perceived efficacy and safety of BZRA, lack of knowledge, work environment and procedure, and ageism. Reported enablers were education, patient motivation, multidisciplinary collaboration and awareness of adverse effects. Although this systematic review was a first step towards BZRA deprescribing enhancement, it only included qualitative studies and was not based on any theoretical framework. Conducting a systematic review, including both qualitative and quantitative evidence, can help further investigate complex processes and systems in health and social care [26]. Moreover, psychological theories can provide a framework for the evaluation of behaviour predictors and to tailor specific interventions [27].

The primary aim of this systematic review was to identify and synthesise barriers and enablers of BZRA deprescribing in older adults and to map them into a theoretical framework. Secondary objectives were the identification of settings or stakeholders for which information is lacking, and the identification of differences in barriers and enablers between different settings.

## Methods

The systematic review protocol was developed based on PRISMA-P guidelines [28] and on the toolkit for mixed-methods studies review by Pluye et al. [29]. It was registered on PROSPERO under the name “Barriers and enablers to benzodiazepine receptor agonists deprescribing” (CRD42020213035). Report follows the 2020 PRISMA checklist for reporting systematic reviews (See additional file 1) [28].

We conducted a systematic search of qualitative, quantitative and mixed-methods studies exploring the views of different stakeholders on BZRA deprescribing in older adults. Five electronic databases were searched from their inception until October 13, 2020: MEDLINE, EMBASE, PsychINFO, CINHAL and CENTRAL. The search strategy was developed with the help of a medical librarian and focused on the three aspects of our question: (i) the studied population, i.e., adults over 65 and their formal and informal caregivers; (ii) the measurement, i.e., perceived barriers and enablers for the studied phenomenon; and (iii) BZRA deprescribing. For each of these aspects, a list of synonyms was constructed with the aim of being as sensitive as possible. Search terms were then combined into a research equation (See additional file 2) that we transcribed for each database. To supplement the database search, we hand-searched the 100 first hits on Google Scholar for grey literature. References from included papers and those citing included papers were also checked for eligibility.

Search results were introduced into the reference management software Endnote X8©, Clarivate Analytics, Philadelphia. Two reviewers (PE and CP) independently screened titles and abstracts for eligibility, using the systematic reviews web application Rayyan [30]. The full texts of potentially eligible studies were read before a final decision was made regarding their eligibility. Disagreements were resolved through discussion with a third reviewer (AS). We included empirical studies using quantitative (interventional or observational), qualitative or mixed-methods designs and published in the English language. Relevant reviews were not included, but their reference lists were checked for studies that had been missed in the database search. Studies assessing barriers and enablers of psychotropic deprescribing in older adults were only included if data on BZRA were presented separately, and then, only these data were extracted. Qualitative studies on BZRA prescribing were only included if they addressed factors related to deprescribing, and only these data were then extracted. We excluded studies with only abstracts available, studies conducted on populations with less than 75% of patients aged over 65 (or caregivers of this population), and studies conducted in populations with specific psychiatric disorders (except for dementia), receiving palliative care, or mentioned as being at the end of life. Eligibility criteria were pilot tested by the two reviewers on a sample of 20 studies. Results of the pilot test were discussed with a third reviewer (AS), and criteria were further adjusted.

The quality of the included studies was independently assessed by two reviewers (PE and CP), using the Mixed-Methods Appraisal Tool (MMAT), version 2018 [31]. The MMAT was selected because it enables quality assessment for all three study types (qualitative, quantitative and mixed-methods). We did not exclude any study based on quality assessment, but results are taken into consideration in the discussion section.

One reviewer (PE) extracted the data from the included studies, using an extraction form that had been previously pilot tested by three reviewers (PE, CP and AS) on one qualitative, one quantitative and one mixed-methods study. A second reviewer (CP) then checked data extraction for accuracy. Extracted data included participants’ quotations from qualitative studies and identified themes underlying BZRA deprescribing, quantitative findings from surveys or questionnaires, and the authors’ conclusions. Predictors of and factors associated with BZRA deprescribing in quantitative studies (interventional or observational) were also extracted. Data extraction form is available in additional file 3.

The theoretical domains framework (TDF), used for data analysis, is a framework that can be used to classify the different determinants of a behaviour [32]. It is particularly relevant for the evaluation of barriers and enablers of performing a behaviour [33]. The validated TDF version 2 encompasses 84 theoretical constructs, divided into 14 domains: Knowledge; Skills; Social/Professional Role and Identity; Beliefs about Capabilities; Optimism; Beliefs about Consequences; Reinforcement; Intentions; Goals; Memory, Attention and Decision Processes; Environmental Context and Resources; Social Influences; Emotions; and Behavioural Regulation [34].

We performed data analysis using a data-based convergent design [35], meaning that results from different study designs were analysed together using a single method, here qualitative. Quantitative results were therefore coded as qualitative data with regard to their interpretation. First, two independent coders (PE and CP), who received training on TDF use, deductively coded the extracted data into TDF domains. Coding disagreements were resolved through discussion and intervention of a third coder (AS). To ease data management, we used NVivo© software, QSR international, Boston. Second, one researcher (PE) identified the most relevant TDF domains for BZRA deprescribing based on the three criteria proposed by Atkins et al.: (i) frequency of the belief, (ii) presence of conflicting beliefs and (iii) perceived importance of the belief [33]. This selection of relevant domains was then checked by a second researcher (CP) for accuracy, and results were discussed within the research team.

## Results

### Search

**Fig. 1:**
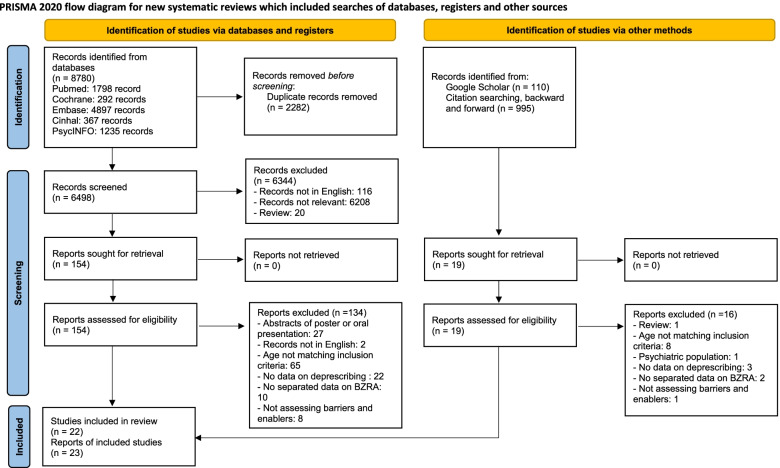
Flow chart of studies screening and selection. *Adapted From:* Page MJ, McKenzie JE, Bossuyt PM, Boutron I, Hoffmann TC, Mulrow CD, et al. The PRISMA 2020 statement: an updated guideline for reporting systematic reviews. BMJ 2021;372:n71. doi: 10.1136/bmj.n71

The electronic database search identified 8780 records. After removing duplicates, 6498 records were screened, and 153 examined in further detail. Of these, we included 20 reports in the present study. We also identified one report through Google Scholar and two through citation searching. Overall, we included 23 reports, from 22 studies (Fig. 1).

### Study characteristics

Of the 23 studies, there were 13 quantitative, 8 qualitative and 2 mixed-methods studies. Two studies reflected on the point of views of different stakeholders from the same sample. The most studied setting was ambulatory, reported in 14 records. NH and hospital settings were explored in 8 and 1 records, respectively. The points of view and/or characteristics of patients, GPs and nurses were assessed in 19, 9 and 3 reports, respectively. A summary of study characteristics and extracted results is provided in Table 1. For 7 of the 23 reports, the data were missing to assess the quality of at least one of the MMAT criteria, and for 2 of these studies, the data were missing for 2 or more criteria. The quality of the majority of the included studies was good, but 2 studies [40, 54] were of poor quality (see Additional file 4).

**Table 1:** Description of characteristics of included studies, significant results and related TDF domains

Author, year	Country	Type of study	Population	Measures/themes explored	BZRA deprescribing definition	Main extracted results
***Studies conducted in the NH setting***						
Anthierens, 2009 [36]	Belgium	Qualitative	33 NH nurses, in 5 NHs	Focus group and face-to-face interviews exploring BZRA use in NH, perceived role of nurses and their attitudes and feelings	Not reported	Different themes and subthemes: Nurses individual attitudes and perceptions: -Routine approach -Nurse as ‘sleep guardian’ Pharmacological knowledge Organisational factors: -Traditional doctor-nurse relationship -Organisational requirements -Smooth running of the NH Team meetings
Bourgeois, 2014 Study A [37]	Belgium	Quantitative, BZRA deprescribing feasibility study, uncontrolled	135 NHRs in 5 NHs	GPs and NHRs willingness to deprescribe Feasibility of deprescribing	BZRA discontinuation	Willingness for discontinuation: GPs refused deprescribing for 71 NHRs Reasons: - Unmotivated patient: 40 - Previous attempt failed: 13 - Other reasons (too old): 9 - Medical cause of sleeplessness: 8 - BZRA not indicated for sleep problems: 6 - Psychiatric history: 5 - Discontinuation started before inclusion possibility: 4 - Stopped BZD but switched to something else: 2 - Discontinuation but no agreement to start in study: 2 13 NHRs out of 51 refused Main reason: reluctance towards change Feasibility of discontinuation: Of 38 NHRs, 66.0% were successful in completely discontinuing BZRA use at 8 months The reasons why NHRs were not successful in their discontinuation was because of sleep problems during the discontinuation (*n*=6/13) and overall loss of motivation (*n*= 5/13)
Bourgeois, 2014 Study B [38]	Belgium	Quantitative cross-sectional study: GP and nurse questionnaire	25 GPs and 16 nurses in 5 NHs	Initiation, indication and previous attempts to stop chronic BZRA use Benefit and harm of BZRA use Willingness to stop BZRA use Resident-specific barriers General attitudes towards BZRA discontinuation	BZRA discontinuation	Previous attempts The GPs and the nurses indicated that they had already attempted a withdrawal in the past in 26% and 12% of the residents, respectively In general, chronic BZRA use was perceived as effective, and only few adverse effects were noted Willingness to stop GPs and nurses indicated willingness to stop chronic BZRA use in respectively 33 and 21% of NHRs Resident-specific barriers most common barriers among the GPs were: -Fear of resistance from the resident -Preference of a pharmacological treatment above a non-pharmacological treatment -Fear that in these resident initial problems would come back -Fear of an increase in the care burden for the staff -Perception that change is not necessary as long as the resident functions well -Fear of withdrawal effects. Among the nurses, the most common barriers were: -Fear that the residents’ initial problems would come back -Preference for a pharmacological treatment -Conviction that change is not necessary as long as the resident functions well. General attitudes The most common attitudes among both GPs and nurses were: -The longer the resident takes the medication, the more difficult it is to stop. -Old age of a resident makes it difficult and unnecessary to stop. -Help of other care professions, such as a psychologist and a pharmacist, are not really necessary. -Alternative strategies are more time-consuming -Rhythm of a nursing home with strict bedtimes also limits possibilities for discontinuation. Nurses agreed and GPs disagreed on the statements that there is little knowledge on alternative strategies to cope with problems when stopping BZDs and that there is little scientific information available for stopping.
de Souto Barreto, 2015 [13]	France	Quantitative non-randomised controlled trial of implementation of an intervention (not targeted at BZRA)	3973 NHRs from 163 NHs, 2167 included in this substudy	Factors associated with BZRA deprescribing at 18 months	BZRA discontinuation	In general, no association for NH and NHR factors BUT living in a particular NH affected BZRA deprescribing In the intervention group, use of an antidepressant was a facilitator and female gender was a barrier In the control group, use of meprobamate and a higher number of medications were both barriers
Evrard, 2020 [39]	Belgium	Quantitative cRCT of an intervention (not targeted at BZRA) implementation	418 NHRs with BZRA at baseline, in 54 NHs	Factors associated with BZRA deprescribing at 15 months	Deprescribing included complete cessation or decreased daily dose and cessation of an “if needed” BZRA prescription in addition to an unchanged chronic dose	Enablers: -Intervention (consisting of education programme for HCPHCPPs, multidisciplinary work and medication review) -Hospitalisation in the past 3 months -Parkinson/extrapyramidal syndrome -Dementia -Number of beds in the NH Barriers: -Public NH (as compared to private non-profit)
Lambson, 2003 [40]	South Africa	Mixed-methods (qualitative interview and quantitative questionnaire)	Interviews: 44 older adults of a retirement village Questionnaire: 10 GPs 4 nursing staff	Interview: reasons for initiation and continuation of BZRA, duration and frequency of use, perception of their doctor’s attitude and prescribing behaviour, desire and/or efforts to stop taking the tablets, and perceptions of usefulness Questionnaire: BZRA perceptions and concerns	Not reported	Patient interviews: -Reasons to continue taking the medication related to fear of not sleeping without them -47% of subjects felt that their doctors were quite happy for them to continue taking the benzodiazepine -26% reported that they would like to stop taking BZRA GP questionnaire: -66% believed that it was acceptable to allow an elderly patient to continue a benzodiazepine indefinitely, and 78% felt that a regular sleeping tablet was a good idea. -44.4% did not mind renewing prescriptions for benzodiazepines, while 33.3% did mind. -55.5% felt it was easier to renew the prescription than to argue with the patient. -79% agreed that patients taking sleeping tablets would not be persuaded to give them up. -67% felt they were bothered by endless requests for benzodiazepine prescriptions Nursing staff questionnaire: -75% felt it was not a problem to continue the sleeping tablet indefinitely -All subjects believed that it was better to tranquillise a restless patient than to allow them to disturb other patients.
MacLagen, 2020 [41]	Canada	Quantitative, retrospective cohort study	35 169 NHRs from Ontario	Factors associated with BZRA discontinuation (in general and stratified by sex)	≥ 30-day gap in days supplied during the 180-day follow-up period after admission	In general Barriers: -Female gender -Low income -Aggregated diagnosis group (more comorbidities) Enablers: -Older age -Higher aggressive behaviour scale score Among men Enablers: -Older age -Widowed -Higher aggressive behaviour scale score Among women Barriers: -Low income -Aggregated diagnosis group (more comorbidities) Enablers: -Older age -Higher aggressive behaviour scale score
Mestres Gonzalvo, 2018 [42]	Netherlands	Quantitative, BZRA deprescribing clinical alert feasibility study, uncontrolled	GPs received 180 alerts about 161 NHRs, in 15 NHs	Reasons for not following a clinical alert related to BZRA deprescribing (as indicated by GPs) Differences between followers and non-followers	Following the clinical rule meant that the GP started BZRA deprescribing	27 out of 161 (16.8%) of the alerts actioned were followed: Reasons for not following: -Already tried (*n*=10, 6.2%) -Patient/family resistance (*n*=37, 23.0%) -No need (non-continuous BZRA use) (*n*=32, 19.9%) -Indication still present (*n*=27, 16.8%) The alert-following group had a shorter median-time of BZRA, compared to the alert non-following group Rate of alert following differed among GPs
***Studies conducted in the ambulatory setting***						
Allary, 2020 [43]	Canada	Quantitative, RCT of gradual withdrawal programme with or without cognitive-behavioural therapy	60 patients of the PASSE-60 study,	Predictors of successful BZRA deprescribing at the patient level in a 16-week RCT, at end of withdrawal, 3 months and 12 months later	Complete BZRA discontinuation	At end of withdrawal: 2 enablers: -Higher social support satisfaction -Lower BZRA dose at baseline At 12 months: 4 enablers -Higher support satisfaction -Self-perceived competence -Higher intensity of depressive symptoms -Poorer quality of sleep
Barter, 1996 [44]	United Kingdom	Qualitative	11 older adults, chronic BZRA users	Interviews about: type of benzodiazepine used; length of use and pattern of taking; social support; reasons for first using these tablets and current reasons; perception of doctor’s attitude and prescribing behaviour; wishes and efforts to stop taking the tablets; and general sleep quality	Not reported	Major themes: -Reported efficacy of the prescribing sleeping tablets -Changes in dosage/pattern of use (for current benzodiazepines) -Reasons for use -Patient knowledge and perception of doctor’s attitude and prescribing behaviour in relation to benzodiazepine use -Wishes, efforts and past experiences of discontinuing tablets -How people felt about using sleeping tablets
Bell, 2011 [45]	Finland	Quantitative, longitudinal (2 years) observational study	311 patients using BZRA	Factors associated with BZRA deprescribing	No benzodiazepine prescription in the last 6 months	One enabler: -Age over 85 years
Chen, 2010 [46]	Canada	Qualitative	13 HCPs working in a geriatric day hospital 5 patients who had been referred to the pharmacist for BZRA deprescribing	Providers: Interviews and group discussion of perceived and actual role in BZRA deprescribing, barriers and enablers, factors that predispose, enable and reinforce Patients: Barriers and facilitators, factors that predispose, enable and reinforce, role of various providers	Not reported	Providers: -Physicians, nurses and pharmacists were the most involved -Other providers lack of guidance Patients, main themes: -Experiences with BZRA -Willingness to taper -Tolerance of tapering
Chen, 2014 [47]	USA	Quantitative, quasi experimental comparative study. Intervention=loosing BZRA reimbursement	Intervention: 250 older adults Control: 216 older adults	Factors associated with BZRA cessation after losing coverage, two regression models	BZRA discontinuation (2 regression models used)	In one of the two models used: Barriers: -Older age -Higher comorbidity scores -Higher BZRA exposure -Higher BZRA dosage Enabler: -Gender: woman
Cook, 2007 Study A [48]	USA	Qualitative	50 older adults, chronic BZRA user	Interviews about: the rationale and circumstances for BZRA use; patient’s perceptions of family members and physicians’ perspectives; knowledge of adverse effects; experience of skipped doses; reliance on benzodiazepines; thoughts about discontinuation; interest in finding alternatives	Not reported	Different themes and subthemes: Purpose and importance of benzodiazepines -Means to cope with stress/anxiety and aid sleep -Lifeline or life-transforming properties -Lack of awareness, underestimation or disregard for adverse effects Attitudes towards tapering/discontinuation -Negativity or resistance to tapering/discontinuation -Rejection of psychological interventions Power and influence of physician-patient relationship
Cook, 2007 Study B [49]	USA	Quantitative, questionnaire	46 older adults taking BZRA for at least 3 months	Factors associated with willingness to taper/discontinue BZRA	Tapering or discontinuation	2 barriers identified in multivariate analysis: -More frequent benzodiazepine intake -Higher Anxiety Sensitivity Index
Cook, 2007 Study C [50]	USA	Qualitative	33 GPs	Interviews about: -Role of BZRA and management of anxiety, insomnia, and depression in older adults -BZRA prescribing and renewing process -Problems with BZRA use and strategies, including psychotherapy use	Not reported	Different themes and subthemes: Physician minimisation of the problem: -No addiction seen in this population -Little recognition of adverse effects other than addiction -Continuation is compassionate, discontinuation is harsh -Low priority relative to medical problems Justification of short-term and long-term BZRA use: -Effectiveness for anxiety and sleep problems -Belief that stable dosage is safe and effective -Attempt to discontinue will fail -Anticipated resistance from patient -Cost-benefit: Question patient gain and highlight suffering involved in tapering or discontinuation Broad organizational factors and system constraints: -Limited physician time -Poor reimbursement for mental health care -Older patients limited acceptance and access to mental health services
Iliffe, 2004 [51]	United Kingdom	Qualitative	192 patients, long-term (>6 months) BZRA users	Interviews about: -BZRA prescribing patterns -Reasons for initial and current prescription -Belief in BZRA efficacy -Concerns about BZRA - Previous discontinuation attempts - Perceived advantages and disadvantages of deprescribing Evaluate differences between continuers and withdrawers	Withdrawers= patients who wished to participate in a withdrawal programme	Reason for prescription: No difference between continuers and withdrawers in reason for initial or current prescription Beliefs of efficacy: -Continuers reported BZRA as more helpful than withdrawers -More continuers reported that they never had sleeping problems while taking BZRA, while withdrawers reported having problems very often Concerns about BZRA: -In both groups the majority of patients reported that no one ever suggested their BZRA may be harmful -Only few patients had worried about long-term use adverse effects, but more withdrawers than continuers said they had worried Perceived advantages and disadvantages of BZRA deprescribing: -No difference between continuers and withdrawers regarding disadvantages (mainly not sleeping and not being able to manage) -Withdrawers were more likely to mention specific benefits, including ‘clearer thinking’, ‘better memory’, ‘being more in control’, ‘more natural sleep’, ‘having to take less tablets’, ‘feeling less sleepy’, ‘feeling proud of myself’ Intention: Withdrawers were more willing than continuers to stop BZRA.
			83 practice staff: 72 GPs, 5 managers, 4 nurses, 2 counsellors	Group interview on main problems, advantages and disadvantages they anticipated in withdrawing their elderly patients from BZRA		Prevalence of beliefs about BZRA deprescribing advantages and disadvantages: -Increased demand on the GP: 48% -Anticipated difficulty in persuading older adults: 51% -Problems anticipated: °Upset or anxious patients: 34% °Patient insomnia: 24% °Unmasked depression: 21% -Expected benefits: °Fewer falls: 57% °Better sleep: 25% °Better quality of life: 18% °Increased independence and unmasking of depression: 11% -Better clinical practice: 65% -Reduced prescription costs: 34% -Addressing a “significant public health issue”: 22% In addition, respondents were concerned about “how patient would react to being encouraged to withdraw from ‘harmful’ drugs which had been prescribed by their own GP”
Joester, 2010 [52]	Australia	Quantitative, retrospective, cross-sectional study	42 BZRA users, over 65, attending a fall clinic	Factors influencing recommendations for BZRA deprescribing Factors associated with compliance	Successful dose reduction or discontinuation	Recommendation to deprescribe BZRA in 31/42 patients Enablers of BZRA deprescribing recommendation: -Assessment by a geriatrician (compared to a rehabilitation specialist) -Patients using BZRA as needed or less than three times per week Compliance with recommendation in 21/28 patients Enabler of recommendation compliance: -Advise to cease BZRA completely (compared with advice to reduce dose or gradually reduce dose with the aim of cessation)
Kuntz, 2018 [53]	USA	Qualitative study, parallel to a direct-to-patient educative intervention	10 older adults, Z-drug users	Interview about: - Past and current Z-drug use - Prior education about sedative - Educational needs - Reactions to the intervention material	Not reported	3 major themes: -Insomnia-related factors (Importance of sleep, treatments alternatives) -Structural and health care delivery system related factor (identification to brochure, individual care, no discussion about it with GP) -Patient experiences and concerns (BZRA taking experience, side-effects)
			7 GPs	Interview about: - Approaches to providing care to older adults with insomnia - Sedative medication prescribing - Reaction to the intervention materials - Factors that hinder or support deprescribing of sedative		3 major themes -Institutional structure (lack of resources, level of priority) -Patient characteristics and attitudes (patient dependence, communication) -Clinician characteristics and attitudes (care burden, alternatives)
Lasserre, 2010 [54]	France	Quantitative: Cross-sectional survey by questionnaire	350 GPs	Questionnaire on: - Knowledge - Opinions about prescriptions - Ways to reduce anxiolytics/hypnotics in older adults - Barriers	Not reported	82% of GPs knew at least one of the 6 national guidelines on management of people with insomnia and/or anxiety 97.1% of GPs previously felt pressure to renew anxiolytics/hypnotics 90.5% declared that it was possible to reduce or stop treatment for their patients High level of patient physical and psychological dependence reported Agreement on ways to reduce anxiolytics/hypnotics: -Campaign to inform general population: 84% -Increased access to psychiatrist: 81% -Reinforcement of physician education: 81% -Increased access to psychotherapy: 60% Agreement with identified barriers: -Patient does not want to stop the treatment: 79% -Psychotherapy is not refunded: 79% -Psychotherapy is not accessible: 73% -No alternative therapy to propose: 70% -Drug withdrawal more dangerous than benefits of stopping: 58% -Relatives of the patient refuse drug withdrawal: 38% -Unaware of drug withdrawal procedure: 8%
Martin, 2017 [55]	Canada	Mixed methods: Quantitative study of factors associated with outcome in the EMPOWER study (educational brochure)	261 adults over 65, receiving at least 5 medicines and chronic BZRA prescription	Evaluation of the three mechanisms of increasing motivation, capacity and opportunity links with outcome	BZRA cessation Individuals who achieved a dose reduction were classified as intent to deprescribe with failed discontinuation	Barriers: -Higher perceived necessity score -Poor health Enablers: -Improved knowledge -Increased concern -Higher risk perception about BZRA -Higher self-efficacy to discontinue
		Qualitative interviews	21 adults over 65, receiving at least 5 medicines and chronic BZRA prescription	Interviews on: - Initial reactions to the intervention - Reasons underlying the decision to taper - Experience with the tapering process - Personal interactions with hcps		Contexts associated with negative outcome (barriers): -Previous discouragement from physician -Poor health status -Unquestioning belief in their physician -Lack of perception of personal risk -Reliance on medication for coping/everyday function -Quality of life focus during end of life -Discouragement from a physician -Intolerance to recurrence of symptoms/withdrawal effects -Loss of confidence to complete the tapering process (post intervention) Contexts associated with positive outcome (enablers): -Previous support from physician/positive attitude towards discontinuation -Stable health status -Certainty and confidence about tapering (post intervention) -Positive outlook on ageing -Perception of increased risk -Lack of psychological attachment -Tapering tool provides support -Supportive HCP
Williams, 2016 [56]	Australia	Qualitative	17 older adults with at least 2 nocturnal BZRA prescriptions in the last 6 months	Interviews on: -BZRA initiation -Perception of doctors’ attitudes -Thoughts on stopping BZRA -Awareness of alternative treatments	Not reported	3 major themes explored: -Commencement and continuation of nocturnal BZRA (reasons for use, benefits perceived, previous deprescribing attempts) -Participants’ knowledge of BZRA and alternative options available (knowledge of adverse effects, treatment alternatives) -Attitudes to BZRA use and cessation (perceived GP opinion, willingness to deprescribe)
***Studies conducted in the hospital setting***						
Yokoi, 2014 [57]	USA	Quantitative, retrospective chart review	75 patients on standing BZRA, admitted for at least 14 days	Differences at admission between continuers and withdrawers Factors potentially associated with deprescribing	No BZRA at discharge	Withdrawers were less anxious at admission than continuers Continuers had a better mean orientation score than withdrawers 2 potential enablers for BZRA discontinuation: -Older age -Higher antidepressant dose

*BZRA* benzodiazepine receptor agonist, *cRCT* cluster randomised control trial, *GP* general practioner, *HCP* health care provider, *NH* nursing home, *NHR* nursing home resident, *TDF* theoretical domains framework

### Identified barriers and enablers

**Fig. 2:**
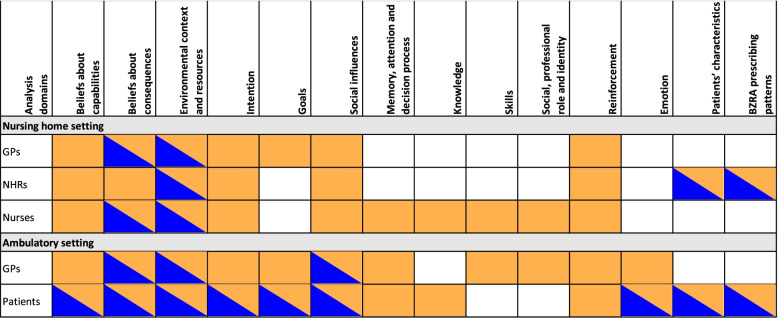
Reported relevant analysis domain, identified as barrier and/or enabler per setting and per stakeholders. Legend: Orange=Barrier, Blue=Enabler, **BZRA**=Benzodiazepine Receptor Agonists, **GPs**=General Practitioners, **NHRs**=Nursing home residents

We identified barriers and enablers across 14 different domains: 7 TDF domains identified as most relevant (beliefs about capabilities; beliefs about consequences; environmental context and resources; intention; goals; social influences; memory, attention and decision process), five other TDF domains, and two domains outside the TDF. The number of domains addressed by the studies ranged from 1 to 11. A summary of the domains identified as barriers and/or enablers per setting and per stakeholder is shown in Fig. 2. We identified mostly barriers. Several domains contained only barriers, whereas others contained barriers and enablers. No domain contained only enablers. Perceived barriers and enablers within domains differed across settings and across stakeholders. As an example, for the domain “beliefs about consequences”, residents in a NH setting reported only barriers, whereas patients in the ambulatory setting and GPs in both settings identified both barriers and enablers. Details of the barriers and enablers identified within each domain per included study are presented in Table 2.

**Table 2:** TDF domains and subthemes identified as barriers and/or enablers in each included study

Qualitative analysis themes	Studies conducted in the NH setting								Studies conducted in ambulatory setting			
	Anthierens, 2009 [36]	Bourgeois, 2014 – study a [37]	Bourgeois, 2014 – study b [38]	de Souto Barreto, 2015 [13]	Evrard, 2020 [39]	Lambson, 2003 [40]	Maclagen, 2020 [41]	Mestres Gonzalvo, 2018 [42]	Allary, 2020 [43]	Barter, 1996 [44]	Bell, 2011 [45]	Chen, 2010 [46]
**Beliefs about capabilities:** ***Perceived capability of stakeholders to perform BZRA deprescribing and the problems they face***												
Patients’ self-efficacy									E			
Deprescribing is challenging			B							B		
Perceived efficacy or lack of efficacy			B			B		B		B;E		
**Beliefs about consequences:*What stakeholders think could happen from performing BZRA deprescribing***												
No perceived benefit			B			B		B		B		B
Return of primary condition		B	B			B						
Withdrawal symptoms			B			B						B
Increase in care burden			B									
Avoiding adverse effects of long-term BZRA			E			E				E		E
**Environmental context and resources:*Influence of the environment on stakeholders’ behaviour***												
Tool implementation					E							E
Favourable/unfavourable moment		B	B							B		
Lack of resources												
Difficulty of alternatives			B									
Heavy workload	B											
Inheritance of prescribing culture												
BZRA deprescribing not prioritised by the healthcare system	B											
NH specific requirements	B		B			B						
NH characteristics				B;E	B;E							
BZRA cost											E	
**Intention:** ***How inclined stakeholders are to perform BZRA deprescribing***												
Level of willingness		B	B			B		B		B		E
No intention to use non-pharmacological approaches			B									
**Goals:*How important is BZRA deprescribing for stakeholders***												
Competing goals			B									
Patients’ attachment to these medicines			B							B		B
Perceived need of sleep	B									E		
Having a more natural sleep										E		E
**Social influences:** ***How others influence stakeholders’ behaviour***												
Expected patient resistance		B	B			B		B				
Pressure for continuous prescribing						B						
Belief that GP’s prescription equals safety and approval for continuous use						B				B		
Patient’s trust in GP										B		
**Memory, attention and decision process:** ***Habits factors and decision process regarding BZRA deprescribing***												
BZRA as an easy solution						B						
Routine approach	B		B			B				B		
Preference for status quo												
**Knowledge:** ***What stakeholders know on BZRA deprescribing***												
Nurses‘ lack of knowledge	B		B									
Patients’ lack of knowledge												B
**Skills:*What stakeholders know about how they should perform BZRA deprescribing***												
GPs’ lack of systematic strategy												
Nurses’ lack of skills regarding non-pharmacological approaches	B											
**Social, professional role and identity:** ***Perception of who stakeholders are (as healthcare professionals)***												
Nurses perceived ideal role	B;E											
Scarce and difficult multidisciplinary work	B		B									B
Perceived expectation of prescribing												
**Reinforcement:** ***Influence of stakeholders’ past experiences with BZRA deprescribing***												
Previous attempts and failure		B	B			B		B		B		
**Emotion:*How stakeholders feel about BZRA deprescribing***												
Various patient emotions												E
GP frustration												
**Patient characteristics**												
Older age			B				E				E	
Woman				B			B					
Depression									E			
Anxiety												
Psychiatric history		B										
Dementia					E							
Aggressive behaviour							E					
Low orientation score												
Parkinson or extrapyramidal symptoms					E							
Comorbidities							B					
Hospitalisation in past 3 months					E							
Higher number of medications				B								
Antidepressant use				E								
Medical cause of sleeplessness		B										
Poorer quality of sleep									E			
Low income							B					
Widowed men							E					
**BZRA prescribing patterns**												
Lower dose									E			
Lower frequency of intake												
Shorter duration of treatment								E				
Indication other than sleeping issues		B										

Qualitative analysis themes	Studies conducted in ambulatory setting										Studies conducted in hospital setting
	Chen, 2014 [47]	Cook, 2007 – study a [48]	Cook, 2007 – study b [49]	Cook, 2007 – study c [50]	Iliffe, 2004 [51]	Joester, 2010 [52]	Kuntz, 2018 [53]	Lasserre, 2010 [54]	Martin, 2017 [55]	Williams, 2016 [56]	Yokoi, 2014 [57]
**Beliefs about capabilities:** ***Perceived capability of stakeholders to perform BZRA deprescribing and the problems they face***											
Patients’ self-efficacy									E		
Deprescribing is challenging		B		B	B		B	B			
Perceived efficacy or lack of efficacy		B		B	B;E		B			B	
**Beliefs about consequences:*What stakeholders think could happen from performing BZRA deprescribing***											
No perceived benefit		B		B	B		B		B	B	
Return of primary condition		B			B		B		B	B	
Withdrawal symptoms				B	B			B			
Increase in care burden					B		B				
Avoiding adverse effects of long-term BZRA		E			E		E		E		
**Environmental context and resources:*Influence of the environment on stakeholders’ behaviour***											
Tool implementation				B			B		E		
Favourable/unfavourable moment							B;E		B;E		
Lack of resources							B	B			
Difficulty of alternatives				B			B	B			
Heavy workload				B			B				
Inheritance of prescribing culture							B				
BZRA deprescribing not prioritised by the healthcare system							B				
NH specific requirements											
NH characteristics							B				
BZRA cost											
**Intention:** ***How inclined stakeholders are to perform BZRA deprescribing***											
Level of willingness		B		B	B	E	B			E	
No intention to use non-pharmacological approaches		B		B							
**Goals:*How important is BZRA deprescribing for stakeholders***											
Competing goals		B		B			B		B		
Patients’ attachment to these medicines		B		B			B	B	B	B	
Perceived need of sleep									E	B	
Having a more natural sleep											
**Social influences:** ***How others influence stakeholders’ behaviour***											
Expected patient resistance				B	B		B	B			
Pressure for continuous prescribing								B			
Belief that GP’s prescription equals safety and approval for continuous use		B							B	B	
Patient’s trust in GP									B;E	E	
**Memory, attention and decision process:** ***Habits factors and decision process regarding BZRA deprescribing***											
BZRA as an easy solution				B							
Routine approach		B					B			B	
Preference for status quo		B									
**Knowledge:** ***What stakeholders know on BZRA deprescribing***											
Nurses‘ lack of knowledge											
Patients’ lack of knowledge					B		B	B	B	B	
**Skills:*What stakeholders know about how they should perform BZRA deprescribing***											
GPs’ lack of systematic strategy				B							
Nurses’ lack of skills regarding non-pharmacological approaches											
**Social, professional role and identity:** ***Perception of who stakeholders are (as healthcare professionals)***											
Nurses perceived ideal role											
Scarce and difficult multidisciplinary work											
Perceived expectation of prescribing				B			B				
**Reinforcement:** ***Influence of stakeholders’ past experiences with BZRA deprescribing***											
Previous attempts and failure		B		B			B			B	
**Emotion:*How stakeholders feel about BZRA deprescribing***											
Various patient emotions		B									
GP frustration				B			B				
**Patient characteristics**											
Older age	B										
Woman	E										
Depression											
Anxiety			B								
Psychiatric history											
Dementia											
Aggressive behaviour											
Low orientation score											E
Parkinson or extrapyramidal symptoms											
Comorbidities	B										
Hospitalisation in past 3 months											
Higher number of medications											
Antidepressant use											E
Medical cause of sleeplessness											
Poorer quality of sleep											
Low income											
Widowed men											
**BZRA prescribing patterns**											
Lower dose	E										
Lower frequency of intake			E			E					
Shorter duration of treatment											
Indication other than sleeping issues											

*BZRA* benzodiazepines receptor agonists, *GP* general practitioner, *NH* nursing home, *TDF* theoretical domains framework [B] stands for an identified barrier and [E] for an identified enabler

Below, we present the data relative to the **TDF domains most relevant** for BZRA deprescribing.

#### Beliefs about capabilities

Beliefs about capabilities represent the perceived capability of stakeholder to perform the behaviour and the problems they face. Twelve studies reported on this domain, mainly as a barrier for BZRA deprescribing; two studies reported that patients’ perceived self-efficacy was an enabler of BZRA deprescribing.

Individuals who decided to deprescribe exhibited higher capacity for tapering, with enhanced self-efficacy compared with those in whom the intervention did not trigger motivation (risk difference, 56.90% (95% CI 45.41% to 65.77%)). Ambulatory setting [55].

GPs and nurses viewed deprescribing, including motivating patients, as challenging.

Nurses considered a stop possible in 21% of the chronic BZD users. NH setting [38]

It can be a tough sell to get patients off of these meds. GP interview, ambulatory setting [53].

Additionally, perceived BZRA efficacy and lack of efficacy were respectively a barrier and an enabler for deprescribing. Studies mainly reported that healthcare providers (HCPs) perceived BZRA as effective, while patients had conflicting views.

More withdrawers reported that their medication was ‘a little helpful’ and more continuers reported that their sleeping tablets were ‘very helpful’ for a good night’s sleep, a significant difference. Ambulatory setting [51].

#### Beliefs about consequences

Beliefs about consequences, which represent what stakeholders think could happen from performing the behaviour, were reported in 13 of the studies, mainly as a barrier. In 11 studies, stakeholders did not perceive any adverse effects of BRZA and therefore did not believe stopping them would have any benefit. As patients aged, healthcare providers and patients themselves believed that BZRA deprescribing would not be beneficial.

I've been on these for so many years and nothing has ever happened so I don’t wanna. Older adult interview, ambulatory setting [48].

Furthermore, some HCPs and patients identified several negative potential consequences of BZRA deprescribing: the return of insomnia or anxiety, withdrawal symptoms and an increase in care burden.

Without this medication, I know that my life would be plagued by anxiety, of this I am certain. (woman, no intent to taper), ambulatory setting [55].

I know that I’m creating a nightmare with follow-up. GP interview, ambulatory setting [53]

However, perception of adverse effects of long-term BZRA, and therefore potential positive effects of deprescribing, was a reported enabler in eight reports.

Answers of a panel of GPs about expected benefits: *“fewer falls (n=47, 57%), better sleep (n=21), better quality of life (n=15, 18%), increased independence and unmasking depression (n=9, 11%). Benefice for the practice itself: 'better clinical practice' (n=47, 65%), reduced prescription costs (n=28, 34%)” Ambulatory setting* [51]*.*

#### Environmental context and resources

Environmental context and resources represent how the environment influence stakeholders’ behaviour. This appeared to be an important domain, reported in 13 of the studies, with barriers and enablers identified at micro (individual and HCP) and macro (system) levels.

At the **micro level**, the implementation of strategies (including multidisciplinary review and education) was reported to be effective in enhancing BZRA deprescribing [39, 55]. Some tools were perceived as helpful, while others sometimes did not reach the GPs or patients or were perceived as inadequate.

In the past I tried to stop the pill all at once. But using the tapering tool, I understood that it need to be a gradual and not a drastic process. (man, successful taper, ambulatory setting) [55]

Guidelines were criticized as out of touch with real-world problems. Ambulatory setting [50].

The environmental context at a patient level may also play an important role, with 5 studies reporting favourable or unfavourable moments in life that may encourage or discourage BZRA deprescribing. These moments might influence the person’s perception of consequences and ability to deprescribe.

Perhaps when I retire, and it is not so important that I go back to sleep, maybe I would consider it then. But right now it seems to serve a purpose. Older adult interview, ambulatory setting [53]

At the **macro level**, studies reported a lack of resources, highlighted by GPs. This is particularly important in a context where GPs and other HCPs have a heavy workload.

Here is the thing: We have infinite resources to prescribe pills. We have very finite and limited resources to actually educate and inform patients about the things they need to know to wean themselves off these medicines. GP interview, ambulatory setting [53]

We do not have enough time for us to follow[-up] these people. We don’t even have time to see our regular patients. GP interview, ambulatory setting [53].

Alternative strategies for treating insomnia and anxiety were few, not available, or not reimbursed. Providing these alternatives was also seen as time consuming and tedious.

Medicare... will not reimburse any Internist for a psychiatric diagnosis. Reimbursement is very low... I think if it was something that we did get reimbursed on I think you would see physicians’ attitudes a lot different. You’d be more willing to spend time. GP interview, ambulatory setting [50].

The GPs and nurses perceived that alternative strategies are more time consuming (median 5 vs. 3, NS). NH setting [38].

In one study, GPs felt that they have inherited the problem from other (older) physicians and a previous prescribing culture.

The problem is, quite frankly, that we don’t start [prescribing] the medication. Most people come in on them. They were given them by their psychiatrist ten years ago and were continued on these medicines, and we are just left with a panel that has a high prevalence [of use] through nothing that I did. GP interview, ambulatory setting [53].

In two studies, BZRA deprescribing was reported to be not prioritised by the healthcare system.

Nobody cares how many patients I have tapered off medication. GP interview, ambulatory setting [53].

Regarding NHs, three studies reported requirements of the specific setting as a barrier. Additionally, being in a specific NH [13] and some NH characteristics (such as private ownership or higher number of beds) [39] were associated with BZRA deprescribing.

The need to have all the residents in bed before the night shift starts and to have the medication round completed enhances BZD use. Nurse interview, NH setting [36].

All subjects believed that it was better to tranquillise a restless patient than to allow them to disturb other patients. study on nurses [40].

Finally, BZRA-associated expenses seem to have a very small role in BZRA deprescribing: only one study highlighted a small association between BZRA cessation and BZRA costs [47].

#### Intention

The intention domain refers to how inclined someone is to perform a specific behaviour. Twelve studies reported various levels of intention for BZRA deprescribing. The overall willingness of patients and HCPs was low.

When asked if they would like to stop taking the benzodiazepine, only 26% (of older adults) felt that they would. NH setting [40].

Reported intention to recommend or use alternatives, including non-pharmacological approaches, was also low.

I just don’t want to. I’m not one of those people who can sit around and talk about my problems with strangers [i.e. cognitive behavioural therapy]. Older adult interview, ambulatory setting [48].

#### Goals

This domain evaluates the importance of the behaviour for stakeholders. We found conflicting results regarding perceived priority of BZRA deprescribing. In five studies, BZRA deprescribing was not reported to be a priority. Indeed, other competing goals were cited by the different stakeholders. Among these, preserving quality of life was more important (five studies). In particular, for older patients, BZRA deprescribing was perceived mainly as impacting one’s well-being near end of life. Treating competing medical issues (three studies) and preserving the patient-doctor relationship (one study) were other goals reported by GPs.

If we have full schedules and only 20 minutes and people have 8 or 9 different problems, and sedative medications is one of them, it is usually not my top priority. GP interview, ambulatory setting [53].

For heaven’s sakes! I’m going to be 91 years old. What difference does it make if you give me something that . . . will hurt me in the future? Older adult interview, ambulatory setting [46].

Nine studies reported strong patient attachment to BZRA, which may reduce the perceived importance of BZRA deprescribing.

Once they find the medication that works, they are very happy and very irritated by any attempts not to prescribe this medication any longer. GP interview, ambulatory setting [53].

Two studies reported the importance of a good night of sleep as a barrier perceived by nurses and patients. However, we also found conflicting results, as two other studies reported that sleep became less important with age [55].

People need to have a good night. It is no use that they lay awake all night and that they are tired the next day and stay in bed all day. Yes I totally agree, sleep is very important in nursing homes, I think even more important than at home. People can be disturbing when they do not sleep… Nurses focus group, NH setting [36].

Nevertheless, we found one enabler in this domain: two studies reported that some patients did not like being on BZRA and wanted to decrease their sleeping pills and have a more natural sleep.

I don’t like being on them, I don’t want to be a slave to something. Older adult interview, ambulatory setting [44].

#### Social influences

Social influence represents how others influence stakeholders’ behaviour. Social support in general [43] and the influence of each possible stakeholder were important determinants of BZRA deprescribing, addressed in 13 of the studies. In particular, studies reported a strong reciprocal influence between GP and patient. Eight studies reported that GPs were afraid of patient resistance or lack of motivation and two studies reported that GPs felt under pressure to renew prescriptions.

Of all eight resident-specific barriers, most common among the GPs were the fear of resistance from the resident (median 9 on 10 points Likert scale). NH setting [38].

Pressure by patients to initiate or renew prescription of anxiolytics/hypnotics had previously been felt by 97.1% of GPs (67.4% often, 29.7% sometimes, 2.9% never). Ambulatory setting [54].

In several studies, patients viewed prescription by the GP as a guarantee that the BZRAs were harmless. Moreover, patients reported that their GP did not inquire about BZRA use and they took this silence as an approval to continue the drugs.

I don’t think (the doctor) is against it ... (the doctor) has never queried it. Older adult interview, ambulatory setting [44].

This finding is reinforced by the great trust patients have in their GP and confidence in their advice that was reported in four studies. As a consequence, studies reported that patients may rely on the GP’s opinion regarding the deprescribing process, which could both be a barrier or an enabler.

I have complete faith in Dr. _____. I mean we go back a lot of years. Whatever he says, goes. Older adult interview, ambulatory setting [48].

To a lesser extent, other forms of social influence were identified: in NHs, nurses reported pressure from colleagues not to attempt any change [36].

#### Memory, attention and decision process

This domain, focusing on habits factors and decision process regarding the studied behaviour, was reported in eight studies, with only barriers for BZRA deprescribing. Two studies reported that GPs perceive BZRA prescribing or continuous prescribing as the easiest solution.

It’s just so much easier to just prescribe something and just walk away. GP interview, ambulatory setting [50].

Seven studies reported a routine approach regarding BZRA use and prescribing for both patients and HCPs: once a BZRA is started, there is a lack of treatment reevaluation.

We do not think enough about sleep medication. People have been taking their sleeping tablets for years. There is no evaluation of whether it is still necessary or not. Nurse interview, NH setting [36].

It is just like putting a comb through your hair, it is just a thing that you are used to. Older adult interview, ambulatory setting [44].

In one study, stakeholder preference for keeping a status quo and reluctance to change was a reported barrier.

The conviction that change is not necessary as long as the resident functions well. Study on GPs and nurses, NH setting [38].

Conflicting attitudes were reported concerning the decision process by patients. On the one hand, they are reported to feel as “critical consumers who weighed the pros and cons of continuing to take nonbenzodiazepines.” [53]. On the other hand, many of them reported not being able to “recall having consulted their doctor with regards to taking a sleeping table” [40] or not considering “how long they would be taking them.” [56].

#### Additional TDF domains

Some relevant barriers and enablers for BZRA deprescribing were also found in **other TDF domains.** For all the subthemes mapped into TDF domains, more citations are available in Additional file 5.

### Knowledge

Studies that investigated HCP knowledge about BZRA deprescribing mainly interrogated GPs and nurses in the NH setting. In that context, GPs were generally aware of BZRA deprescribing recommendations, that BZRA should not be renewed indefinitely, and of the withdrawal procedure [54]. NH nurses acknowledged a lack of knowledge both on benzodiazepines and their adverse effects, and on sleep hygiene and non-pharmacological approaches for anxiety or insomnia management. Among patients, six studies reported very limited knowledge about BZRA adverse effects or alternative therapies. Moreover, an improvement in patient knowledge was associated with BZRA deprescribing [55].

### Skills

One study [50] reported that physicians reported a lack of a systematic strategy to address patient’s concerns regarding deprescribing. For NH nurses, one study reported a lack of skills regarding implementation of non-pharmacological approaches.

### Social, professional role and identity

One study reported that the nurses’ perceived role includes reporting on patients’ sleep habits and looking for solutions [36]. Consequently, they would be helpful in a multidisciplinary process. Yet, this multidisciplinary approach is currently reported as too scarce and nurses often feel they are not listened to by GPs. For GPs, two studies reported that they felt they were expected to give something to help the patient [50, 53].

### Reinforcement

In nine studies, patients and GPs indicated that they had attempted to deprescribe BZRA and failed, which was a barrier to future attempts. However, the link between these previous attempts and future attempts was not observed in all studies [37, 38].

### Emotion

Only four studies reported on stakeholders’ emotions. Although some patients felt fear or anxiety regarding BZRA deprescribing [48], it could also be seen as an unimportant event [46]. For GPs, the process of BZRA deprescribing was reported as frustrating, because of the level of challenge and effort required [50, 53].

Finally, we identified **two other themes** that did not fit into the TDF, as they are not behaviour related. These themes were “patient characteristics” and “BZRA prescribing patterns”.

### Patient characteristics

Many studies identified diverse patient characteristics associated with an increased likelihood of BZRA deprescribing: depression [43], Parkinson’s or extrapyramidal syndrome [39], dementia [39], poorer orientation score [57], aggressive behaviour [41] poorer quality of sleep [43], hospitalisation in the past 3 months [39], antidepressant use [13, 57] and widowed men [41]. Other patient characteristics were associated with a decreased likelihood of BZRA deprescribing: anxiety [49], low income [41], psychiatric history [37], higher comorbidities [41, 47], higher number of medications [13] and medical cause of sleeplessness [37]. For other characteristics, the influence on BZRA deprescribing was inconsistent across studies. Older age was associated with increased [41, 45] and decreased [38, 47] BZRA deprescribing. Some studies reported that deprescribing was higher among women than men [47], but conflicting results were also found [13, 41].

### BZRA prescribing patterns

A few factors were reported as being positively associated with BZRA deprescribing: a lower BZRA dose [43, 47], a lower frequency of BZRA intake [49, 52] and a shorter duration of treatment [42]. BZRA not used for sleeping issues however reported as a barrier for deprescribing [37].

## Discussion

In this systematic review of barriers and enablers for BZRA deprescribing in older adults, we included 23 studies and identified determinants in and out of TDF domains. Compared to another recent systematic review on this topic, which only included qualitative evidence [25], our approach enabled us to include more data and gain a deeper understanding of BZRA deprescribing. Consequently, we were able to report additional barriers and enablers. The use of the TDF is also valuable with regard to the implementation of future strategies.

### Identified barriers and enablers

The most relevant domains were Beliefs about capabilities, Beliefs about consequences, Environmental context and resources, Intention, Goals, Social influences, and Memory, Attention and Decision Process. Most domains were relevant to the ambulatory and NH settings, but there were some specificities to the NH setting (environmental context, role of nurses).

One may wonder whether these results are specific to BZRA deprescribing, or common to deprescribing in general, as it is known that some barriers and enablers might be medication-specific [58]. As an example, BZRA are known to cause physical and psychological dependence, which may impact deprescribing. A systematic review of patient barriers and enablers for deprescribing [59] found the following barriers and enablers that can be linked to our sub-theme analysis: disagreement or agreement with appropriateness of cessation (patients’ lack of knowledge, beliefs about consequences), absence or presence of a process for cessation (tool implementation), negative or positive influences to cease medication (social influences, reinforcement), fear of cessation (beliefs about consequences, emotion) and dislike of medication (attachment to the medicine). Consequently, our results show that these general barriers and enablers also apply to BZRA deprescribing. However, we found additional barriers not reported for general medications, such as the lack of intention to use a non-pharmacological approach, or seeing BZRA as an easy solution.

### Moving forward to implementation

There is a reported need to translate known barriers and enablers into strategies and tool implementation [58]. Using the TDF enables the identified relevant domains to be linked to behavioural change techniques (BCT) [60]. A recent scoping review identified the BCTs implemented in deprescribing strategies conducted in primary health care [61]. They reported a wide range of BCTs, often used in combination. BCTs were mainly mapped into functions of “environmental restructuring”, “enablement” and “persuasion”, which also seem appropriate for some of the barriers and enablers we report. A next step would be to choose from among these BCTs those that are best suited for BZRA deprescribing, based on the results of our systematic review. As an example, the barriers of “no perceived benefit” and “competing goals” could respectively be targeted by the BCTs of salience of consequences and goal setting. Combining these BCTs to create a complex strategy is more likely to be effective. As an example, the EMPOWER study used a patients’ brochure combining different BCTs, information about health consequences and instructions on how to perform a behaviour. The simple use of this brochure led to a 27% reduction in BZRA use [62]. The determinants of BZRA prescribing patterns and patient characteristics could help choose priority groups for future interventions.

One may also wonder whether past strategies have targeted the TDF domains and barriers and enablers that we reported in this systematic review. Some reviews of strategies targeting BZRA deprescribing in older adults [21, 63] or in adults in primary care [20] have reported on the effects of education, gradual dose reduction, use of alternatives, non-pharmacological approaches, tool implementation and medication review. All these strategies are individual-level (micro) strategies and target the following identified barriers: patient lack of knowledge, no perceived benefit, GP lack of systematic strategy, no intention to use non-pharmacological alternatives, difficulty of alternatives and tool implementation. Although some individual barriers and enablers have been targeted, some major behavioural determinants, such as memory, attention and decision processes or social influences domains, still need to be addressed. Moreover, our systematic review also highlighted barriers and enablers that need to be addressed at the healthcare system-level (macro), such as lack of resources. We found one systematic review of the effects of strategies targeting this macro level [64]. These strategies included making BZRA harder to prescribe, withdrawing the driving licence, promoting alternatives through campaigns, increasing the financial burden of BZRA or giving financial incentives to physicians. These policies address barriers that we identified: lack of patient knowledge, BZRA being an easy solution, competing goals and BZRA deprescribing not prioritised by healthcare systems. Nevertheless, further macro-level initiatives are needed.

In the future, implementing a BCT targeting each of the most relevant domains identified in the present review should enhance the probability of success. If possible, strategies should be developed at different levels of the healthcare system to enhance BZRA deprescribing, including the organisational (macro) level. Importantly, as barriers and enablers differ depending on stakeholders and setting, components of the strategies need to be flexible and adapted to account for this and developed in close collaboration with stakeholders.

### Recommendations for future research

Among the 23 included studies, the point of view of some stakeholders was under-evaluated and deserves further exploration. Data specific to deprescribing among older adults with cognitive impairment require further investigation. Moreover, no study included informal caregivers or relatives, although such persons are particularly important in dementia patients, for example. Only one study interviewed pharmacists [46], although pharmacists are often involved in deprescribing strategies. The points of view of psychologists or psychiatrists were also not reported. As these specialists may play an important role in the implementation of non-pharmacological management of insomnia and anxiety, this is a major research gap. Additionally, the hospital setting was under-evaluated in our review, yet it may be an appropriate setting to initiate a deprescribing process.

### Strengths and limitations

This review has several strengths. Firstly, including both qualitative and quantitative evidences enabled a deeper comprehension of the complex BZRA deprescribing phenomenon. Indeed, we were able to include various studies addressing this specific topic from different points of view. Secondly, using the TDF as an analysis guide is valuable for the theoretically informed development of future strategies. Thirdly, we used both deductive and inductive coding. By doing so, we were able to include barriers and enablers that did not fit the TDF and therefore develop a more complete understanding of their determinants.

This review also has several limitations. Firstly, none of the included studies used the TDF. Consequently, we had to code based on inference from the text. The use of the TDF itself was also challenging, as some items potentially fit into several domains. However, we were able to reach agreement between researchers, and asked for help from a specialised researcher when needed, which strengthens the validity of findings. Secondly, we did not include non-English literature. Thirdly, because of the qualitative approach of our analysis, we were not able to evaluate the specific effect of each barrier and enabler. Finally, the included studies were conducted in only nine countries. Therefore, it is likely that some of our results are not transferable to other countries, in particular the barriers and enablers identified in the environmental context and resource domain.

## Conclusion

By systematically reviewing barriers and enablers for BZRA deprescribing, we were able to identify the most relevant TDF domains and other determinants. While similar barriers and enablers were reported across different settings of care, there are also singular barriers at the environmental context level which need to be taken into account. Future investigation should focus on the identified barriers and enablers at macro- and micro-levels, as well as addressing research gaps.

## Supplementary information

**Additional file 1.** PRISMA 2020 Checklist.

**Additional file 2.** Research equations.

**Additional file 3. **Data extraction form.

**Additional file 4.** Quality assessment of studies included in the review.

**Additional file 5.**  TDF domains, analysis subthemes and matching citations. (Contains additional citations for each analysis subtheme).

## Data Availability

The datasets used and/or analysed during the current study are available from the corresponding author on reasonable request.

## Abbreviations

BCT: Behavioural change techniques
BZRA: Benzodiazepine receptor agonists
GP: General practitioner
HCP: Healthcare providers
MMAT: Mixed-methods appraisal tool
NH: Nursing home
TDF: Theoretical domains framework
