# Supplementary material for: Barriers and enablers for deprescribing benzodiazepine receptor agonists in older adults: a systematic review of qualitative and quantitative studies using the theoretical domains framework
Source: Implement Sci. 2022 Jul 8;17:41. doi: 10.1186/s13012-022-01206-7 (PMC9264665; doi:10.1186/s13012-022-01206-7)
Supplement: Supplementary file 2 — Additional file 2. Research equations. [file 13012_2022_1206_MOESM2_ESM.docx]

**Additional file 2:** Search equation for each database

Cochrane :

(("older person"):ti,ab OR ("old person"):ti,ab OR ("older persons"):ti,ab OR ("old persons"):ti,ab OR ("older adult"):ti,ab OR ("older adults"):ti,ab OR ("old patients"):ti,ab OR ("older people"):ti,ab OR ("elderly"):ti,ab OR ("old age"):ti,ab OR ("seniors"):ti,ab OR ("senior person"):ti,ab OR ("senior persons"):ti,ab OR ("senior people"):ti,ab OR ("frail"):ti,ab OR ("frailty"):ti,ab OR ("frailness"):ti,ab OR [mh "aged"] OR ("physician"):ti,ab OR ("physicians"):ti,ab OR ("doctor"):ti,ab OR ("doctors"):ti,ab OR ("general practitioner"):ti,ab OR ("general practitioners"):ti,ab OR ("general practioner"):ti,ab OR ("general practioners"):ti,ab OR [mh "physicians"] OR [mh "general practitioners"] OR ("geriatrician"):ti,ab OR ("geriatricians"):ti,ab OR [mh "geriatricians"] OR ("gerontologist"):ti,ab OR ("gerontologists"):ti,ab OR ("nurse"):ti,ab OR ("nurses"):ti,ab OR [mh "nurses"] OR ("pharmacist"):ti,ab OR ("pharmacists"):ti,ab OR [mh "pharmacists"] OR ("prescriber"):ti,ab OR ("prescribers"):ti,ab OR ("healthcare provider"):ti,ab OR ("healthcare providers"):ti,ab OR ("health personnel"):ti,ab OR [mh "health personnel"] OR ("healthcare professional"):ti,ab OR ("healthcare professionals"):ti,ab OR ("healthcare practitioner"):ti,ab OR ("healthcare practitioners"):ti,ab OR ("caregiver"):ti,ab OR ("caregivers"):ti,ab OR [mh "caregivers"] OR ("carer"):ti,ab OR ("carers"):ti,ab OR ("older patient"):ti,ab OR ("older patients"):ti,ab ) AND (("benzodiazepine"):ti,ab OR ("benzodiazepines"):ti,ab OR [mh "benzodiazepines"] OR ("benzodiazepin"):ti,ab OR ("benzodiazepins"):ti,ab OR ("z-drug"):ti,ab OR ("z-drugs"):ti,ab OR ("anxiolytic"):ti,ab OR ("anxiolytics"):ti,ab OR ("anti-anxiety agent"):ti,ab OR ("anti-anxiety agents"):ti,ab OR [mh "anti-anxiety agents"] OR ("sleeping pill"):ti,ab OR ("sleeping pills"):ti,ab OR [mh "sleep aids, pharmaceutical"] OR ("sleep aid"):ti,ab OR ("sleep aids"):ti,ab OR ("sleeping aid"):ti,ab OR ("sleeping aids"):ti,ab OR ("sleep medication"):ti,ab OR ("sleep medications"):ti,ab OR ("sedative"):ti,ab OR ("sedatives"):ti,ab OR ("hypnotic"):ti,ab OR ("hypnotics"):ti,ab OR [mh "hypnotics and sedatives"] OR ("tranquilizing agent"):ti,ab OR ("tranquilizing agents"):ti,ab OR [mh "tranquilizing agents"] OR ("tranquilizer"):ti,ab OR ("tranquilizers"):ti,ab OR ("hypnosedative"):ti,ab OR ("hypnosedatives"):ti,ab ) AND ([mh "deprescriptions"] OR ("deprescription"):ti,ab OR ("deprescriptions"):ti,ab OR ("deprescribe"):ti,ab OR ("deprescribed"):ti,ab OR ("deprescribing"):ti,ab OR ("stop medicaton"):ti,ab OR ("stop medications"):ti,ab OR ("medication decrease"):ti,ab OR ("drug decrease"):ti,ab OR ("dose decrease"):ti,ab OR ("dosage reduction"):ti,ab OR ("dosage reductions"):ti,ab OR ("dose reduction"):ti,ab OR ("dose reductions"):ti,ab OR ("withdrawal"):ti,ab OR ("withdrawals"):ti,ab OR ("taper"):ti,ab OR ("tapering"):ti,ab OR ("prescribing"):ti,ab OR ("discontinuation"):ti,ab OR ("cessation"):ti,ab OR [mh "withholding treatment"] OR ("withdrawing"):ti,ab ) AND (("attitude"):ti,ab OR ("attitudes"):ti,ab OR [mh "attitude"] OR ("behavior"):ti,ab OR ("behaviors"):ti,ab OR ("behaviour"):ti,ab OR ("behaviours"):ti,ab OR [mh "behavior"] OR ("preferences"):ti,ab OR ("perspectives"):ti,ab OR ("determinants"):ti,ab OR ("barriers"):ti,ab OR ("facilitators"):ti,ab OR ("enabler"):ti,ab OR ("enablers"):ti,ab OR ("experiences"):ti,ab OR ("experience"):ti,ab OR ("perception"):ti,ab OR ("perceptions"):ti,ab OR ("thoughts"):ti,ab OR ("view"):ti,ab OR ("willingness"):ti,ab OR ("predictors"):ti,ab OR ("opinion"):ti,ab OR ("opinions"):ti,ab OR ("belief"):ti,ab OR ("beliefs"):ti,ab OR ("factors"):ti,ab OR [mh "motivation"] OR ("motivation"):ti,ab OR ("motivations"):ti,ab )

Embase

('aged'/exp OR 'physician'/exp OR 'caregiver'/exp OR 'paramedical personnel'/exp OR 'older person':ti,ab OR 'old person':ti,ab OR 'older persons':ti,ab OR 'old persons':ti,ab OR 'older adult':ti,ab OR 'older adults':ti,ab OR 'old patients':ti,ab OR 'older people':ti,ab OR 'elderly':ti,ab OR 'old age':ti,ab OR 'seniors':ti,ab OR 'senior person':ti,ab OR 'senior persons':ti,ab OR 'senior people':ti,ab OR 'frail':ti,ab OR 'frailty':ti,ab OR 'frailness':ti,ab OR 'physician':ti,ab OR 'physicians':ti,ab OR 'doctor':ti,ab OR 'doctors':ti,ab OR 'general practitioner':ti,ab OR 'general practitioners':ti,ab OR 'general practioner':ti,ab OR 'general practioners':ti,ab OR 'geriatrician':ti,ab OR 'geriatricians':ti,ab OR 'gerontologist':ti,ab OR 'gerontologists':ti,ab OR 'nurse':ti,ab OR 'nurses':ti,ab OR 'pharmacist':ti,ab OR 'pharmacists':ti,ab OR 'prescriber':ti,ab OR 'prescribers':ti,ab OR 'healthcare provider':ti,ab OR 'healthcare providers':ti,ab OR 'health personnel':ti,ab OR 'healthcare professional':ti,ab OR 'healthcare professionals':ti,ab OR 'healthcare practitioner':ti,ab OR 'healthcare practitioners':ti,ab OR 'caregiver':ti,ab OR 'caregivers':ti,ab OR 'carer':ti,ab OR 'carers':ti,ab OR 'older patient':ti,ab OR 'older patients':ti,ab ) AND ('z drug'/exp OR 'anxiolytic agent'/exp OR 'benzodiazepine receptor stimulating agent'/exp OR 'hypnotic agent'/exp OR 'benzodiazepine':ti,ab OR 'benzodiazepines':ti,ab OR 'benzodiazepin':ti,ab OR 'benzodiazepins':ti,ab OR 'z-drug':ti,ab OR 'z-drugs':ti,ab OR 'anxiolytic':ti,ab OR 'anxiolytics':ti,ab OR 'anti-anxiety agent':ti,ab OR 'anti-anxiety agents':ti,ab OR 'sleeping pill':ti,ab OR 'sleeping pills':ti,ab OR 'sleep medication':ti,ab OR 'sleep medications':ti,ab OR 'sedative':ti,ab OR 'sedatives':ti,ab OR 'hypnotic':ti,ab OR 'hypnotics':ti,ab OR 'tranquilizing agent':ti,ab OR 'tranquilizing agents':ti,ab OR 'tranquilizer':ti,ab OR 'tranquilizers':ti,ab OR 'hypnosedative':ti,ab OR 'hypnosedatives':ti,ab ) AND ('deprescribing'/exp OR 'deprescription'/exp OR 'stop medicaton':ti,ab OR 'stop medications':ti,ab OR 'medication decrease':ti,ab OR 'drug decrease':ti,ab OR 'dose decrease':ti,ab OR 'dosage reduction':ti,ab OR 'dosage reductions':ti,ab OR 'dose reduction':ti,ab OR 'dose reductions':ti,ab OR 'medication cessation ':ti,ab OR 'medications cessation ':ti,ab OR 'drug cessation':ti,ab OR 'medicine cessation':ti,ab OR 'benzodiazepine cessation':ti,ab OR 'benzodiazepines cessation':ti,ab OR 'Z-drug cessation':ti,ab OR 'z-drugs cessation':ti,ab OR 'medication discontinuation':ti,ab OR 'medications discontinuation':ti,ab OR 'benzodiazepine discontinuation':ti,ab OR 'benzodiazepines discontinuation':ti,ab OR 'withdrawal':ti,ab OR 'withdrawals':ti,ab OR 'taper':ti,ab OR 'tapering':ti,ab OR 'prescribing':ti,ab OR 'discontinuation':ti,ab OR 'cessation':ti,ab OR 'withdrawing':ti,ab ) AND ('behavior'/exp OR 'willingness'/exp OR 'belief'/exp OR 'beliefs'/exp OR 'experience'/exp OR 'predictor variable'/exp OR 'predictors'/exp OR 'attitude':ti,ab OR 'attitudes':ti,ab OR 'behavior':ti,ab OR 'behaviors':ti,ab OR 'behaviour':ti,ab OR 'behaviours':ti,ab OR 'preferences':ti,ab OR 'perspectives':ti,ab OR 'determinants':ti,ab OR 'barriers':ti,ab OR 'facilitators':ti,ab OR 'enabler':ti,ab OR 'enablers':ti,ab OR 'experiences':ti,ab OR 'experience':ti,ab OR 'perception':ti,ab OR 'perceptions':ti,ab OR 'thoughts':ti,ab OR 'views':ti,ab OR 'view':ti,ab OR 'willingness':ti,ab OR 'predictors':ti,ab OR 'opinion':ti,ab OR 'opinions':ti,ab OR 'belief':ti,ab OR 'beliefs':ti,ab OR 'factors':ti,ab OR 'motivation':ti,ab OR 'motivations':ti,ab )

Pubmed

("older person"[tiab] OR "old person"[tiab] OR "older persons"[tiab] OR "old persons"[tiab] OR "older adult"[tiab] OR "older adults"[tiab] OR "old patients"[tiab] OR "older people"[tiab] OR "elderly"[tiab] OR "old age"[tiab] OR "seniors"[tiab] OR "senior person"[tiab] OR "senior persons"[tiab] OR "senior people"[tiab] OR "frail"[tiab] OR "frailty"[tiab] OR "frailness"[tiab] OR "aged"[mh] OR "physician"[tiab] OR "physicians"[tiab] OR "doctor"[tiab] OR "doctors"[tiab] OR "general practitioner"[tiab] OR "general practitioners"[tiab] OR "general practioner"[tiab] OR "general practioners"[tiab] OR "physicians"[mh] OR "general practitioners"[mh] OR "geriatrician"[tiab] OR "geriatricians"[tiab] OR "geriatricians"[mh] OR "gerontologist"[tiab] OR "gerontologists"[tiab] OR "nurse"[tiab] OR "nurses"[tiab] OR "nurses"[mh] OR "pharmacist"[tiab] OR "pharmacists"[tiab] OR "pharmacists"[mh] OR "prescriber"[tiab] OR "prescribers"[tiab] OR "healthcare provider"[tiab] OR "healthcare providers"[tiab] OR "health personnel"[tiab] OR "health personnel"[mh] OR "healthcare professional"[tiab] OR "healthcare professionals"[tiab] OR "healthcare practitioner"[tiab] OR "healthcare practitioners"[tiab] OR "caregiver"[tiab] OR "caregivers"[tiab] OR "caregivers"[mh] OR "carer"[tiab] OR "carers"[tiab] OR "older patient"[tiab] OR "older patients"[tiab] ) AND ("benzodiazepine"[tiab] OR "benzodiazepines"[tiab] OR "benzodiazepines"[mh] OR "benzodiazepin"[tiab] OR "benzodiazepins"[tiab] OR "z-drug"[tiab] OR "z-drugs"[tiab] OR "anxiolytic"[tiab] OR "anxiolytics"[tiab] OR "anti-anxiety agent"[tiab] OR "anti-anxiety agents"[tiab] OR "anti-anxiety agents"[mh] OR "sleeping pill"[tiab] OR "sleeping pills"[tiab] OR "sleep aids, pharmaceutical"[mh] OR "sleep aid"[tiab] OR "sleep aids"[tiab] OR "sleeping aid"[tiab] OR "sleeping aids"[tiab] OR "sleep medication"[tiab] OR "sleep medications"[tiab] OR "sedative"[tiab] OR "sedatives"[tiab] OR "hypnotic"[tiab] OR "hypnotics"[tiab] OR "hypnotics and sedatives"[mh] OR "tranquilizing agent"[tiab] OR "tranquilizing agents"[tiab] OR "tranquilizing agents"[mh] OR "tranquilizer"[tiab] OR "tranquilizers"[tiab] OR "hypnosedative"[tiab] OR "hypnosedatives"[tiab] ) AND ("deprescriptions"[mh] OR "deprescription"[tiab] OR "deprescriptions"[tiab] OR "deprescribe"[tiab] OR "deprescribed"[tiab] OR "deprescribing"[tiab] OR "stop medicaton"[tiab] OR "stop medications"[tiab] OR "medication decrease"[tiab] OR "drug decrease"[tiab] OR "dose decrease"[tiab] OR "dosage reduction"[tiab] OR "dosage reductions"[tiab] OR "dose reduction"[tiab] OR "dose reductions"[tiab] OR "withdrawal"[tiab] OR "withdrawals"[tiab] OR "taper"[tiab] OR "tapering"[tiab] OR "prescribing"[tiab] OR "discontinuation"[tiab] OR "cessation"[tiab] OR "withholding treatment"[mh] OR "withdrawing"[tiab] ) AND ("attitude"[tiab] OR "attitudes"[tiab] OR "attitude"[mh] OR "behavior"[tiab] OR "behaviors"[tiab] OR "behaviour"[tiab] OR "behaviours"[tiab] OR "behavior"[mh] OR "preferences"[tiab] OR "perspectives"[tiab] OR "determinants"[tiab] OR "barriers"[tiab] OR "facilitators"[tiab] OR "enabler"[tiab] OR "enablers"[tiab] OR "experiences"[tiab] OR "experience"[tiab] OR "perception"[tiab] OR "perceptions"[tiab] OR "thoughts"[tiab] OR "view"[tiab] OR "willingness"[tiab] OR "predictors"[tiab] OR "opinion"[tiab] OR "opinions"[tiab] OR "belief"[tiab] OR "beliefs"[tiab] OR "factors"[tiab] OR "motivation"[mh] OR "motivation"[tiab] OR "motivations"[tiab] )

PsycInfo

(TIAB( "older person" ) OR TIAB( "old person" ) OR TIAB( "older adult" ) OR TIAB( "older adults" ) OR TIAB( "old patients" ) OR TIAB( "older people" ) OR TIAB( "elderly" ) OR TIAB( "old age" ) OR TIAB( "seniors" ) OR TIAB( "senior person" ) OR TIAB( "senior persons" ) OR TIAB( "senior people" ) OR TIAB( "frail" ) OR TIAB( "frailty" ) OR TIAB( "frailness" ) OR MESH( "aged" ) OR TIAB( "physician" ) OR TIAB( "physicians" ) OR TIAB( "doctor" ) OR TIAB( "doctors" ) OR TIAB( "general practitioner" ) OR TIAB( "general practitioners" ) OR MESH( "physicians" ) OR MESH( "general practitioners" ) OR TIAB( "geriatrician" ) OR TIAB( "geriatricians" ) OR MESH( "geriatricians" ) OR TIAB( "gerontologist" ) OR TIAB( "gerontologists" ) OR TIAB( "nurse" ) OR TIAB( "nurses" ) OR MESH( "nurses" ) OR TIAB( "pharmacist" ) OR TIAB( "pharmacists" ) OR MESH( "pharmacists" ) OR TIAB( "prescriber" ) OR TIAB( "prescribers" ) OR TIAB( "healthcare provider" ) OR TIAB( "healthcare providers" ) OR TIAB( "health personnel" ) OR MESH( "health personnel" ) OR TIAB( "healthcare professional" ) OR TIAB( "healthcare professionals" ) OR TIAB( "healthcare practitioner" ) OR TIAB( "healthcare practitioners" ) OR TIAB( "caregiver" ) OR TIAB( "caregivers" ) OR MESH( "caregivers" ) OR TIAB( "carer" ) OR TIAB( "carers" ) OR TIAB( "older patient" ) OR TIAB( "older patients" ) OR SU.EXPLODE( "aged" ) OR SU.EXPLODE( "Older Adulthood" ) OR SU.EXPLODE( "Aging" ) OR SU.EXPLODE( "Aging (attitudes toward)" ) OR SU.EXPLODE( "Health personnel" ) OR SU.EXPLODE( "geriatric patients" ) OR SU.EXPLODE( "Elder care" ) OR SU.EXPLODE( "Caregivers" ) ) AND (TIAB( "benzodiazepine" ) OR TIAB( "benzodiazepines" ) OR MESH( "benzodiazepines" ) OR TIAB( "benzodiazepin" ) OR TIAB( "benzodiazepins" ) OR TIAB( "z-drug" ) OR TIAB( "z-drugs" ) OR TIAB( "anxiolytic" ) OR TIAB( "anxiolytics" ) OR TIAB( "anti-anxiety agent" ) OR TIAB( "anti-anxiety agents" ) OR MESH( "anti-anxiety agents" ) OR TIAB( "sleeping pill" ) OR TIAB( "sleeping pills" ) OR MESH( "sleep aids, pharmaceutical" ) OR TIAB( "sleep aid" ) OR TIAB( "sleep aids" ) OR TIAB( "sleeping aid" ) OR TIAB( "sleeping aids" ) OR TIAB( "sleep medication" ) OR TIAB( "sleep medications" ) OR TIAB( "sedative" ) OR TIAB( "sedatives" ) OR TIAB( "hypnotic" ) OR TIAB( "hypnotics" ) OR MESH( "hypnotics and sedatives" ) OR TIAB( "tranquilizing agent" ) OR TIAB( "tranquilizing agents" ) OR MESH( "tranquilizing agents" ) OR TIAB( "tranquilizer" ) OR TIAB( "tranquilizers" ) OR TIAB( "hypnosedative" ) OR TIAB( "hypnosedatives" ) OR SU.EXPLODE( "Benzodiazepines" ) OR SU.EXPLODE( "Tranquilizing drugs" ) OR SU.EXPLODE( "Hypnotic drugs" ) OR SU.EXPLODE( "Sedatives" ) ) AND (MESH( "deprescriptions" ) OR TIAB( "deprescription" ) OR TIAB( "deprescriptions" ) OR TIAB( "deprescribe" ) OR TIAB( "deprescribed" ) OR TIAB( "deprescribing" ) OR TIAB( "stop medicaton" ) OR TIAB( "stop medications" ) OR TIAB( "medication decrease" ) OR TIAB( "drug decrease" ) OR TIAB( "dose decrease" ) OR TIAB( "dosage reduction" ) OR TIAB( "dosage reductions" ) OR TIAB( "dose reduction" ) OR TIAB( "dose reductions" ) OR TIAB( "withdrawal" ) OR TIAB( "withdrawals" ) OR TIAB( "taper" ) OR TIAB( "tapering" ) OR TIAB( "prescribing" ) OR TIAB( "discontinuation" ) OR TIAB( "cessation" ) OR MESH( "withholding treatment" ) OR TIAB( "withdrawing" ) OR SU.EXPLODe( "Drug withdrawal" ) ) AND (TIAB( "attitude" ) OR TIAB( "attitudes" ) OR MESH( "attitude" ) OR TIAB( "behavior" ) OR TIAB( "behaviors" ) OR TIAB( "behaviour" ) OR TIAB( "behaviours" ) OR MESH( "behavior" ) OR TIAB( "preferences" ) OR TIAB( "perspectives" ) OR TIAB( "determinants" ) OR TIAB( "barriers" ) OR TIAB( "facilitators" ) OR TIAB( "enabler" ) OR TIAB( "enablers" ) OR TIAB( "experiences" ) OR TIAB( "experience" ) OR TIAB( "perception" ) OR TIAB( "perceptions" ) OR TIAB( "thoughts" ) OR TIAB( "view" ) OR TIAB( "willingness" ) OR TIAB( "predictors" ) OR TIAB( "opinion" ) OR TIAB( "opinions" ) OR TIAB( "belief" ) OR TIAB( "beliefs" ) OR TIAB( "factors" ) OR MESH( "motivation" ) OR TIAB( "motivation" ) OR TIAB( "motivations" ) OR SU.EXPLODE( "Awareness" ) OR SU.EXPLODE( "Client attitudes " ) OR SU.EXPLODE( "Health attitudes" ) OR SU.EXPLODE( "Health personnel attitudes" ) OR SU.EXPLODE( "Behavior" ) OR SU.EXPLODE( "Motivation" ) OR SU.EXPLODE( "Experiences (Events)" ) OR SU.EXPLODE( "Predictability" ) )

Cinhal

(MH "aged" OR MH "caregivers" OR MH "general practitioners" OR MH "geriatricians" OR MH "health personnel" OR MH "nurses" OR MH "pharmacists" OR MH "physicians" OR TI "caregiver" OR TI "caregivers" OR TI "carer" OR TI "carers" OR TI "doctor" OR TI "doctors" OR TI "elderly" OR TI "frail" OR TI "frailness" OR TI "frailty" OR TI "general practioner" OR TI "general practioners" OR TI "general practitioner" OR TI "general practitioners" OR TI "geriatrician" OR TI "geriatricians" OR TI "gerontologist" OR TI "gerontologists" OR TI "health personnel" OR TI "healthcare practitioner" OR TI "healthcare practitioners" OR TI "healthcare professional" OR TI "healthcare professionals" OR TI "healthcare provider" OR TI "healthcare providers" OR TI "nurse" OR TI "nurses" OR TI "old age" OR TI "old patients" OR TI "old person" OR TI "old persons" OR TI "older adult" OR TI "older adults" OR TI "older patient" OR TI "older patients" OR TI "older people" OR TI "older person" OR TI "older persons" OR TI "pharmacist" OR TI "pharmacists" OR TI "physician" OR TI "physicians" OR TI "prescriber" OR TI "prescribers" OR TI "senior people" OR TI "senior person" OR TI "senior persons" OR TI "seniors" OR AB "caregiver" OR AB "caregivers" OR AB "carer" OR AB "carers" OR AB "doctor" OR AB "doctors" OR AB "elderly" OR AB "frail" OR AB "frailness" OR AB "frailty" OR AB "general practioner" OR AB "general practioners" OR AB "general practitioner" OR AB "general practitioners" OR AB "geriatrician" OR AB "geriatricians" OR AB "gerontologist" OR AB "gerontologists" OR AB "health personnel" OR AB "healthcare practitioner" OR AB "healthcare practitioners" OR AB "healthcare professional" OR AB "healthcare professionals" OR AB "healthcare provider" OR AB "healthcare providers" OR AB "nurse" OR AB "nurses" OR AB "old age" OR AB "old patients" OR AB "old person" OR AB "old persons" OR AB "older adult" OR AB "older adults" OR AB "older patient" OR AB "older patients" OR AB "older people" OR AB "older person" OR AB "older persons" OR AB "pharmacist" OR AB "pharmacists" OR AB "physician" OR AB "physicians" OR AB "prescriber" OR AB "prescribers" OR AB "senior people" OR AB "senior person" OR AB "senior persons" OR AB "seniors" ) AND (MH "anti-anxiety agents" OR MH "benzodiazepines" OR MH "hypnotics and sedatives" OR MH "sleep aids, pharmaceutical" OR MH "tranquilizing agents" OR TI "anti-anxiety agent" OR TI "anti-anxiety agents" OR TI "anxiolytic" OR TI "anxiolytics" OR TI "benzodiazepin" OR TI "benzodiazepine" OR TI "benzodiazepines" OR TI "benzodiazepins" OR TI "hypnosedative" OR TI "hypnosedatives" OR TI "hypnotic" OR TI "hypnotics" OR TI "sedative" OR TI "sedatives" OR TI "sleep aid" OR TI "sleep aids" OR TI "sleep medication" OR TI "sleep medications" OR TI "sleeping aid" OR TI "sleeping aids" OR TI "sleeping pill" OR TI "sleeping pills" OR TI "tranquilizer" OR TI "tranquilizers" OR TI "tranquilizing agent" OR TI "tranquilizing agents" OR TI "z-drug" OR TI "z-drugs" OR AB "anti-anxiety agent" OR AB "anti-anxiety agents" OR AB "anxiolytic" OR AB "anxiolytics" OR AB "benzodiazepin" OR AB "benzodiazepine" OR AB "benzodiazepines" OR AB "benzodiazepins" OR AB "hypnosedative" OR AB "hypnosedatives" OR AB "hypnotic" OR AB "hypnotics" OR AB "sedative" OR AB "sedatives" OR AB "sleep aid" OR AB "sleep aids" OR AB "sleep medication" OR AB "sleep medications" OR AB "sleeping aid" OR AB "sleeping aids" OR AB "sleeping pill" OR AB "sleeping pills" OR AB "tranquilizer" OR AB "tranquilizers" OR AB "tranquilizing agent" OR AB "tranquilizing agents" OR AB "z-drug" OR AB "z-drugs" ) AND (MH "deprescriptions" OR MH "withholding treatment" OR TI "cessation" OR TI "deprescribe" OR TI "deprescribed" OR TI "deprescribing" OR TI "deprescription" OR TI "deprescriptions" OR TI "discontinuation" OR TI "dosage reduction" OR TI "dosage reductions" OR TI "dose decrease" OR TI "dose reduction" OR TI "dose reductions" OR TI "drug decrease" OR TI "medication decrease" OR TI "prescribing" OR TI "stop medications" OR TI "stop medicaton" OR TI "taper" OR TI "tapering" OR TI "withdrawal" OR TI "withdrawals" OR TI "withdrawing" OR AB "cessation" OR AB "deprescribe" OR AB "deprescribed" OR AB "deprescribing" OR AB "deprescription" OR AB "deprescriptions" OR AB "discontinuation" OR AB "dosage reduction" OR AB "dosage reductions" OR AB "dose decrease" OR AB "dose reduction" OR AB "dose reductions" OR AB "drug decrease" OR AB "medication decrease" OR AB "prescribing" OR AB "stop medications" OR AB "stop medicaton" OR AB "taper" OR AB "tapering" OR AB "withdrawal" OR AB "withdrawals" OR AB "withdrawing" ) AND (MH "attitude" OR MH "behavior" OR MH "motivation" OR TI "attitude" OR TI "attitudes" OR TI "barriers" OR TI "behavior" OR TI "behaviors" OR TI "behaviour" OR TI "behaviours" OR TI "belief" OR TI "beliefs" OR TI "determinants" OR TI "enabler" OR TI "enablers" OR TI "experience" OR TI "experiences" OR TI "facilitators" OR TI "factors" OR TI "motivation" OR TI "motivations" OR TI "opinion" OR TI "opinions" OR TI "perception" OR TI "perceptions" OR TI "perspectives" OR TI "predictors" OR TI "preferences" OR TI "thoughts" OR TI "view" OR TI "willingness" OR AB "attitude" OR AB "attitudes" OR AB "barriers" OR AB "behavior" OR AB "behaviors" OR AB "behaviour" OR AB "behaviours" OR AB "belief" OR AB "beliefs" OR AB "determinants" OR AB "enabler" OR AB "enablers" OR AB "experience" OR AB "experiences" OR AB "facilitators" OR AB "factors" OR AB "motivation" OR AB "motivations" OR AB "opinion" OR AB "opinions" OR AB "perception" OR AB "perceptions" OR AB "perspectives" OR AB "predictors" OR AB "preferences" OR AB "thoughts" OR AB "view" OR AB "willingness" )

Google Scholar

filetype:pdf ("general practitioners" OR "healthcare providers" OR "older adults") (anxiolytics OR benzodiazepines OR hypnotics OR sedatives) (deprescribing OR deprescription OR discontinuation) (attitudes OR barriers OR beliefs OR enablers)
