# Supplementary material for: Barriers and enablers for deprescribing benzodiazepine receptor agonists in older adults: a systematic review of qualitative and quantitative studies using the theoretical domains framework
Source: Implement Sci. 2022 Jul 8;17:41. doi: 10.1186/s13012-022-01206-7 (PMC9264665; doi:10.1186/s13012-022-01206-7)
Supplement: Supplementary file 4 — Additional file 4. Quality assessment of studies included in the review. [file 13012_2022_1206_MOESM4_ESM.docx]

| **Table 1a. Quality assessment of the qualitative studies included in the review** | | | | | |
| --- | --- | --- | --- | --- | --- |
|  | **Questions from the Mixed Method Appraisal Tool (MMAT), version 2018** | | | | |
| **Included studies** | Is the qualitative approach appropriate to answer the research question? | Are the qualitative data collection methods adequate to address the research question? | Are the findings adequately derived from the data? | Is the interpretation of results sufficiently substantiated by data? | Is there coherence between qualitative data sources, collection, analysis and interpretation? |
| Anthierens, S. (2009) | Yes | Yes | Yes | Yes | Yes |
| Barter, G (1996) | Yes | Yes | Can’t tell | Yes | Yes |
| Chen, L.  (2010) | Yes | Yes | Yes | Yes | Yes |
| Cook, J – A (2007) | Yes | Yes | Yes | Yes | Yes |
| Cook, J. – C (2007) | Yes | Yes | Yes | Yes | Yes |
| Illiffe, S.  (2004) | Yes | Yes | Yes | Yes | No |
| Kuntz, J. (2018) | Yes | Yes | Yes | Yes | Yes |
| Williams, F. (2016) | Yes | Yes | Yes | Yes | Yes |

**Additional file 4: Quality assessment of studies included in the review**

| **Table 1b. Quality assessment of the quantitative randomized controlled trials included in the review** | | | | | |
| --- | --- | --- | --- | --- | --- |
|  | **Questions from the Mixed Method Appraisal Tool (MMAT), version 2018** | | | | |
| **Included studies** | Is randomization appropriately performed? | Are the groups comparable at baseline? | Are there complete outcome data? | Are outcome assessors blinded to the intervention provided? | Did the participants adhere to the assigned intervention? |
| Allary, A.  (2020) | Yes | Can’t tell | Yes | Can’t tell | Can’t tell |
| Evrard, P.  (2020) | Yes | Yes | Yes | Yes | Can’t tell |

| **Table 1c. Quality assessment of the quantitative non-randomized studies included in the review** | | | | | |
| --- | --- | --- | --- | --- | --- |
|  | **Questions from the Mixed Method Appraisal Tool (MMAT), version 2018** | | | | |
| **Included studies** | Are the participants representative of the target population? | Are measurements appropriate regarding both the outcome and intervention (or exposure)? | Are there complete outcome data? | Are the confounders accounted for in the design and analysis? | During the study period, is the intervention administered (or exposure occurred) as intended? |
| Chen, YC.  (2014) | Yes | Yes | Yes | Yes | Yes |
| De Souto Barreto, P.  (2015) | Yes | Yes | Yes | Yes | Can’t tell |

| **Table 1d. Quality assessment of the quantitative descriptive studies included in the review** | | | | | |
| --- | --- | --- | --- | --- | --- |
|  | **Questions from the Mixed Method Appraisal Tool (MMAT), version 2018** | | | | |
| **Included studies** | Is the sampling strategy relevant to address the research question? | Is the sample representative of the target population? | Are the measurements appropriate? | Is the risk of nonresponse bias low? | Is the statistical analysis appropriate to answer the research question? |
| Bell, J.  (2011) | Yes | Yes | Yes | Yes | Yes |
| Bourgeois, J – A (2014) | Yes | Yes | Yes | Yes | Yes |
| Bourgeois, J – B (2014) | Yes | Can’t tell | Yes | Yes | Yes |
| Cook, J – B (2007) | Yes | Yes | Yes | Yes | Yes |
| Joester, J.  (2010) | Yes | Can’t tell | Yes | Can’t tell | Yes |
| Lasserre, A.  (2009) | Yes | No | Yes | No | Yes |
| MacLagen, L.  (2020) | Yes | Yes | Yes | Yes | Yes |
| Mestres Gonzalvo, C.  (2018) | Yes | Yes | Yes | Yes | No |
| Yokoi, Y.  (2014) | Yes | Yes | Yes | Yes | Yes |

| **Table 1e. Quality assessment of the mixed-methods studies included in the review** | | | | | |
| --- | --- | --- | --- | --- | --- |
|  | **Questions from the Mixed Method Appraisal Tool (MMAT), version 2018, for the qualitative part** | | | | |
| **Included studies** | Is the qualitative approach appropriate to answer the research question? | Are the qualitative data collection methods adequate to address the research question? | Are the findings adequately derived from the data? | Is the interpretation of results sufficiently substantiated by data? | Is there coherence between qualitative data sources, collection, analysis and interpretation? |
| Martin, P.  (2017) | Yes | Yes | Yes | Yes | Yes |
| Lambson, MA.  (2003) | Yes | Yes | Can’t tell | No | No |
|  | **Questions from the Mixed Method Appraisal Tool (MMAT), version 2018, for the quantitative part** | | | | |
|  | Is the sampling strategy relevant to address the research question? | Is the sample representative of the target population? | Are the measurements appropriate? | Is the risk of nonresponse bias low? | Is the statistical analysis appropriate to answer the research question? |
| Martin, P.  (2017) | Yes | Yes | Yes | Yes | Yes |
| Lambson, MA.  (2003) | Yes | Can’t tell | Can’t tell | No | Yes |
|  | **Questions from the Mixed Method Appraisal Tool (MMAT), version 2018, regarding mixed method design** | | | | |
|  | Is there an adequate rationale for using a mixed methods design to address the research question? | Are the different components of the study effectively integrated to answer the research question? | Are the outputs of the integration of qualitative and quantitative components adequately interpreted? | Are divergences and inconsistencies between quantitative and qualitative results adequately addressed? | Do the different components of the study adhere to the quality criteria of each tradition of the methods involved? |
| Martin, P.  (2017) | Yes | Yes | Yes | Yes | Yes |
| Lambson, MA.  (2003) | Yes | Can’t tell | Can’t tell | No | Yes |
