# Supplementary material for: Barriers and enablers for deprescribing benzodiazepine receptor agonists in older adults: a systematic review of qualitative and quantitative studies using the theoretical domains framework
Source: Implement Sci. 2022 Jul 8;17:41. doi: 10.1186/s13012-022-01206-7 (PMC9264665; doi:10.1186/s13012-022-01206-7)
Supplement: Supplementary file 5 — Additional file 5. TDF domains, analysis subthemes and matching citations. (Contains additional citations for each analysis subtheme). [file 13012_2022_1206_MOESM5_ESM.docx]

| **Additional file 5: TDF domains, analysis subthemes and matching citations** | | |
| --- | --- | --- |
| TDF domain and definition | Analysis  Sub-themes | Extracted citations in included studies |
| Knowledge  *What stakeholders know on BZRA deprescribing* | Nurses’ lack of knowledge | “*P1 Sleeping pills do not give that many side effects. P2: well apart from the fall incidents. P3 But I think those fall incidents are more associated with the mobility of the older in general, it is not directly linked with sleeping pills. (general agreement in the group) P4 We don’t really know what the side effects are, maybe incontinence because they are asleep they do not realise that they have to got to the bathroom…*” Focus group of nurses, nursing home setting [1]  *“Nurses agreed and GPs disagreed on the statements that there is little knowledge on alternative strategies to cope with problems when stopping benzodiazepines (median 7 vs. 2, p < 0.001) and that there is little scientific information available for stopping (medians 6 vs. 2, p = 0.004*).” Nursing home setting [2] |
|  | Patient’s lack of knowledge | *“No not that I am aware of.” –* Female, 73 years of age */ “Probably get hypnotised I suppose. No I don’t really know of anything”. –* Female, 79 years of age, ambulatory setting [3]  *“Study participants* (older adults) *in the majority of cases were unaware of alternative treatments available for insomnia.”* Ambulatory setting *[3]*  *“GPs felt that more information should be distributed to the general population (84%)* Ambulatory setting *[4]* |
| Skills  *What stakeholders know about how they should perform BZRA deprescribing* | GPs’ lack of systematic strategy | “*None had a systematic strategy for addressing patient concerns that inevitably emerge during this process*.” Study about GP, ambulatory setting [5] |
|  | Nurses lack of skill regarding the implementation of non-pharmacological approaches | “*At the moment when residents can’t sleep, the only solution we know is to give them sleep medication. I do know that certain techniques exist like relaxation therapy or aromatherapy. I know these things exist but I don’t know that much about it, I must say*” Interview of a nurse, nursing home setting [1] |
| Social, professional role & identity  *Perception of who stakeholders are (as healthcare professionals)* | Nurses perceived ideal role | *“Person1: I do think we have a role in BZD use, we can influence the physician. I do think our story plays an important role. Person2: Yes, definitely, we have to report if a patient has slept well or has slept badly and then the physician will prescribe or not prescribe accordingly.”* Focus group of nurses, nursing home setting [1]  *“Actually as a nurse we would have to look for the cause of the problem instead of immediately giving a sleeping pill. In fact, we should look for a solution, but in reality…*” Interview of a nurse, nursing home setting [1] |
|  | Scarce and difficult multidisciplinary work | *“It is all a little ‘non-interdisciplinary’, I mean: we should have a moment that we all come together, however, there are hardly any other professionals at those meeting”* Interview of a nurse, nursing home setting [1]  *“We should evaluate our residents more often, but if you feel like you have no say in the decision…you can try and talk to the doctor and tell him wouldn’t it be better to try and withdraw that patient from BZD, but if every time the doctors says: if she is happy with her sleeping pill…then you know there is nothing more that you can do…”* interview of a nurse, nursing home setting *[1]* |
|  | Perceived expectation of prescribing | *“You feel like you’ve gotta give the patient something to help.”* Interview of a GP, ambulatory setting [5] |
| Beliefs about capabilities  *Perceived capability of stakeholders to perform BZRA deprescribing and the problems they face* | Patients’ self-efficacy | *“Individuals who decided to deprescribe exhibited higher capacity for tapering, with enhanced self-efficacy compared with those in whom the intervention did not trigger motivation (risk difference, 56.90% (95% CI 45.41% to 65.77%))”* Ambulatory setting [6] |
|  | Deprescribing is challenging | “*Nurses considered a stop possible in 21% of the chronic BZD users.*” Nursing home setting [2]  “*It can be a tough sell to get patients off of these meds.”* Interview of a GP, ambulatory setting [6] |
|  | Perceived efficacy or lack of efficacy | *“GPs and nurses indicated that the BZD still had the desired effect in respectively 87% and 83% of the chronic BZD users.”* Nursing home setting [2]  *“[Zolpidem] works, and that’s what I want to stay on*” Interview of an older adult, ambulatory setting [7]  *“More withdrawers reported that their medication was ‘a little helpful’ and more continuers reported that their sleeping tablets were ‘very helpful’ for a good night’s sleep, a significant difference. More continuers reported that they never had sleeping problems after taking their nightly benzodiazepine, whereas more withdrawers reported that they had problems ‘very often’, again a significant difference”* ambulatory setting [8]  “*I take one and a half because they are not very strong and one is not quite enough." "I had to increase to two because I wasn't getting enough sleep."* Interviews of older adults, ambulatory setting [9]  *“I don’t get a full night’s sleep with them*”. – Female, 86 years of age, ambulatory setting [3] |
| Beliefs about consequences  *What stakeholders think could happen from performing BZRA deprescribing* | No perceived benefit | *“ I’ve been on these for so many years and nothing has ever happened so I don’t wanna."* Interview of older patient, ambulatory setting [10]  *“Physicians reasoned that a benzodiazepine at a stable dose for months–years was probably helping and doing no harm.”* Ambulatory setting [5] |
|  | Return of primary condition | *“Without this medication, I know that my life would be plagued by anxiety, of this I am certain”.* 68-year-old woman, no intent to taper, ambulatory setting [6]  *“Well if I don’t take them, I literally don’t get any sleep.”* – Female, 86 years, ambulatory setting [3]  *"Oh, I think I would jump out of my skin." "I’d probably be screamin’ all the time."* Interview of older adults, ambulatory setting [10] |
|  | Withdrawal symptoms | “*Drug withdrawal more dangerous than benefits of stopping: 58% (of GPs) agree, completely agree”* Nursing home setting [11]  “*physicians believed that reducing or discontinuing benzodiazepines would unnecessarily cause undue suffering.”* Ambulatory setting [5] |
|  | Increase in care burden | *“I know that I’m creating a nightmare with follow-up*.” Interview of a GP, ambulatory setting [7] |
|  | Avoiding side-effects of long-term BZRA | *“When asked about the main advantages of stopping taking sleeping pills continuers were much more likely to say ‘none’ or ‘don’t know’ while withdrawers were more likely to mention specific benefits such as ‘clearer thinking’, ‘being more in control’ and ‘better memory’. Withdrawers were also more likely to suggest additional benefits including, ‘having to take less tablets’, ‘feeling less sleepy’, ‘more natural sleep’, ‘feeling proud of myself’. Group differences in responses were significant.”* Ambulatory setting [7]  Answers of a panel of GPs about exepected benefits: *“fewer falls (n=47, 57%) better sleep (n=21) better quality of life (n=15, 18%) increased independance and unmasking depression (n=9, 11%) Benefice for the practice itself: 'better clinical practice' (n=47, 65%), reduced prescription costs (n=28, 34%)”* Ambulatory setting [7] |
| Reinforcement  *Influence of stakeholders’ past experiences with BZRA deprescribing* | Previous attempts and failure | *“(The doctor) did suggest that I come off them, which I did for a month but I was quite ill. I could not sleep . . . and then (the doctor) suggested I go on temazepam*.” Interview of odler patient, ambulatory setting [9]  “*When the Mandrax was taken away it nearly killed me*” Interview of older patient, ambulatory setting [9]  “*The GPs and the nurses indicated that they already attempted a withdrawal in the past in respectively 26% and 12% of the residents*” Nursing home setting [2] |
| Intention  *How inclined stakeholders are to perform BZRA deprescribing* | Level of willingness | *“I’m not gonna experiment with myself, not at this age.*” Interview of older patient, ambulatory setting [10]  *“Only in 13% of the residents, the GP and the nurse were both willing to stop”* Ambulatory setting [2]  “*When asked if they would like to stop taking the benzodiazepine, only 26% (of older adults) felt that they would*” Nursing home setting [11] |
|  | No intention to use non-pharmacological approaches | “*For many of the patients, there was an explicit rejection of psychological intervention either to treat their underlying distress or to assist in taper/ discontinuation.”* Ambulatory setting [9]  *"I don’t think counseling’s gonna help me at this stage of my life."* Interview of an older adult, ambulatory setting [9]  *"I just don’t want to. I’m not one of those people who can sit around and talk about my problems with strangers."* Interview of an older adult, ambulatory setting [9] |
| Goals  *How important is BZRA deprescribing for stakeholders* | Competing goals | *“If we have full schedules and only 20 minutes and people have 8 or 9 different problems, and sedative medications is one of them, it is usually not my top priority*” Interview of a GP, ambulatory setting [6]  *“I don’t think it’s worth getting into a big knock-down drag-out [fight] with them or having them leave my practice over this issue.”* Interview of a GP, ambulatory setting [4]  “*I can’t lose patients over this*” Interview of a GP, ambulatory setting [4]  *“For heaven’s sakes! I’m going to be 91 years old. What difference does it make if you give me something that . . . will hurt me in the future? How long do you think my future is?”* Interview of older patient, ambulatory setting [12] |
|  | Patient’s attachment to the medicine | *“They (the patients) explained that benzodiazepines had an important function in their lives, ranging from soothing properties providing peace and emotional control to miraculously extending life. For the most part, patients saw benzodiazepines not as a life-enhancing luxury but as restorative and necessary to maintain a normal life, and anticipated that life without benzodiazepines would be of a decidedly lower quality.”* Ambulatory setting [10]  *"I just can’t do it. Are you trying to tell me that I should stop or not take it? I’d be miserable. Are you gonna tell him [the physician] not to prescribe it anymore? Please don’t tell him not to give it to me." "Don’t you dare take that stuff away from me."* Interview of older patients, ambulatory setting [10]  *“Once they find the medication that works, they are very happy and very irritated by any attempts not to prescribe this medication any longer.”* Interview of a GP, ambulatory setting [7]  *“I think they enjoy the effects of the benzodiazepine and they don’t want to have that taken away. It’s literally like taking candy from a baby and people that have enjoyed the effects of that class of drugs don’t wanna give it up”.* Interview of a GP, ambulatory setting [5] |
|  | Perceived need of sleep | “*People need to have a good night. It is no use that they lay awake all night and that they are tired the next day and stay in bed all day. Yes I totally agree, sleep is very important in nursing homes, I think even more important than at home. People can be disturbing when they do not sleep…*” Focus group of nurses, nursing home setting [1]  “*At my age I don’t believe in miracles such as being able to sleep for 8, 9 or 10 hours each night. It would be impossible for me, so I content myself with the hours of sleep I get*” 84-year-old man, successful taper, ambulatory setting [6] |
|  | Having a more natural sleep | *“Most patients wanted to decrease their use of medications, to give up sleeping pills and to get a better, “more natural” sleep.”* Ambulatory setting [9]  *“I don't like being on them, I don't want to be a slave to something.”* Interview of older adult, ambulatory setting [9] |
| Memory, attention and decision process  *Habits factors and decision process regarding BZRA deprescribing* | BZRA as an easy solution | “*It’s just so much easier to just prescribe something and just walk away.”* Interview of a GP, ambulatoru setting [5]  “*A benzodiazepine becomes a quick fix because you don’t have time, this is what theywant, theydon’t feel good, here it is. It numbs them up and you’re not gonna get a phone call afterwards, you’re not gonna get anything, you’ll see them in a month, here’s your renewal, see ya later*.” Interview of a GP, ambulatory setting [5] |
|  | Routine approach | “*It’s just so much easier to just prescribe something and just walk away.”* Interview of a GP, ambulatory setting [5]  “*A benzodiazepine becomes a quick fix because you don’t have time, this is what theywant, theydon’t feel good, here it is. It numbs them up and you’re not gonna get a phone call afterwards, you’re not gonna get anything, you’ll see them in a month, here’s your renewal, see ya later*.” Interview of a GP, ambulatory setting [5] |
|  | Preference for status quo | *“the conviction that change is not necessary as long as the resident functions well”* barrier reported in a study on GPs and nurses, nursing home setting [2]  *"If it works I’m not touching it. I don’t think there would be anything better."* Interview of an older adult, ambulatory setting *[10]* |
| Environmental context and resources  *How the environment influences stakeholders’ behaviour* | Tools implementation | *“In the past I tried to stop the pill all at once. But using the tapering tool, I understood that it need to be a gradual and not a drastic process”.* (84-year-old man, successful taper, ambuuulatory setting) *[6]*  *“guidelines were criticized as out of touch with real-world problems”* Ambulatory setting *[5]*  *“The brochure didn’t target me at all. There are many different kinds of insomnia. … I mean, people who don’t really have insomnia think there is only one kind.”* Interview of older patient, ambulatory setting *[7]*  *“It is going to take more than a pamphlet. …”* Interview of a GP, ambulatory setting *[7]* |
|  | Favorable or unfavorable moment | *“Perhaps when I retire, and it is not so important that I go back to sleep, maybe I would consider it then. But right now it seems to serve a purpose.”* Interview of older patients, ambulatory setting [7]  “*I don’t have as much pain as I used to. It’s now under control so it was easier for me to stop. Before—no way*”. 68-year-old woman, successful taper, ambulatory setting [6] |
|  | Lack of resources | *“Here is the thing: We have infinite resources to prescribe pills. We have very finite and limited resources to actually educate and inform patients about the things they need to know to wean themselves off these medicines. The limiting factor is not only what patients are willing to do but also the resources that we can provide them to help out.”* Interview of a GP, ambulatory setting [7]  *“The only thing I really strongly recommend is that we have good support. We can start the taper off, but if I could send the patient information to a certain pool and tell them that they have to monitor a patient for four weeks to see how she is doing.”* Interview of a GP, ambulatory setting *[6]*  *“[W]e need more resources to help people with insomnia.”* Interview of a GP, ambulatory setting *[6]*  *“GPs agreed that greater access to psychiatrists could help to reduce prescription of psychotropic drugs”* Ambulatory setting *[12]* |
|  | Difficulty of alternatives | *“Medicare... will not reimburse any Internist for a psychiatric diagnosis. Reimbursement is very low... I think if it was something that we did get reimbursed on I think you would see physicians’ attitudes a lot different. You’d be more willing to spend time.”* Interview of a GP, ambulatory setting *[5]*  *“I think patients in my office are much more ready to admit the fact that taking this medicine now is cheaper and simpler and easier and less stigma, nobody knows about, etcetera and [patients think]”* Interview of a GP, ambulatory *setting [5]*  *“The GPs and nurses perceived that alternative strategies are more time consuming (median 5 vs. 3, NS)”* Nursing home setting *[2]* |
|  | Heavy workload | *“Even though that would give them more job satisfaction, they feel that they are not working in an ‘ideal setting’ and they are often overwhelmed by work pressure, which stimulates medicalisation.”* Study about nurses, nursing home setting *[1]*  *“We do not have enough time for us to follow[-up] these people. We don’t even have time to see our regular patients”* Interview of a GP, ambulatory setting [6] |
|  | Inheritance of prescribing culture | *“The problem is, quite frankly, that we don’t start [prescribing] the medication. Most people come in on them. They were given them by their psychiatrist ten years ago and were continued on these medicines, and we are just left with a panel that has a high prevalence [of use] through nothing that I did”* Interview of a GP, ambulatory setting *[6]* |
|  | BZRA deprescribing not prioritized by the healthcare system | *“Nobody cares how many patients I have tapered off medication.”* Interview of a GP, ambulatory setting *[6]*  *“Maybe we should make that a point of interest within our institution.”* Interview of a nurse, nursing home setting *[1]*  *“Primary care clinicians suggested a need for the health care system to prioritize deprescribing, present tapering as an activity prioritized by the system rather than only a single clinician, and to create incentives that would encourage the clinician to deprescribe.”* Ambulatory setting *[6]* |
|  | NH specific requirement | *“The need to have all the residents in bed before the night shift starts and to have the medication round completed enhances BZD use.”* Nursing home setting [1]  *“Person 1: Well, you have to wake them up to give them their sleeping pill (laughter in the group). Person2: You can laugh, but it is reality, we do after all have to sign the medication list.”* Focus group of nurses, nursing home setting [1]  *“All subjects believed that it was better to tranquillise a restless patient that to allow them to disturb other patients”* Nursing home setting [11] |
| Social influences  *How others influence stakeholders’ behaviour* | Expected patient’s resistance | *“Of all eight resident-specific barriers, most common among the GPs were the fear of resistance from the resident (median 9 on 10 point Likert scale)”* Nursing home setting [2] |
|  | Pressure for continuous prescribing | *“Pressure by patients to initiate or renew prescription of anxiolytics/hypnotics had previously been felt by 97.1% of GPs (67.4% often, 29.7% sometimes, 2.9% never).”*Ambulatory setting [13]  *“Every time I tried to say Lorazepam isn’t the right medication, she said, ‘but I’ve been on it for thirty years.”* Interview of a GP, ambulatory setting [4] |
|  | Belief that GPs prescription equal safety and approval for continuous use | “*"I don't think they (sleeping pills) hurt me very much, because when I ask for them I get a fair amount." "I don't think (the doctor) is against it .. . (the doctor) has never queried it.*"” Interviews of older adults, ambulatory setting [9]  *"He [the physician] wouldn’t have given it to me if he thought it was gonna hurt me."* Interview of an older adult, ambulatory setting [10]  *“Well she doesn’t say anything. It didn’t worry her she just wrote the script*. – Female, 73 years of age” ambulatory setting [3]. |
|  | Patient’s trust in GP | "*I have complete faith in Dr. _____. I mean we go back a lot of years. What ever he says, goes."* Interview of an older adult, ambulatory setting [9]  *‘If you take all your pills as prescribed, you’ll never have problems in your life […] When my doctor prescribes something for me, I know it’s not junk, I know it’s good for me. And I don’t question it’*. 72-year-old man, no intent to taper, ambulatory setting [5]  *“If the doctor said to me I don’t want you to take it anymore … I wouldn’t take them”*. – Male, 67 years of age, ambulatory setting [3] |
| Emotion  How stakeholders feel about BZRA deprescribing | Various patients’ emotions | *"I think I will shriek out if anybody took it away because I know that Dr. ____ wanted to put me on something else. Well, I thought I was gonna space out. Oh, man, I was sittin’ here shakin’."* Interview of an older adult, ambulatory setting [10]  “*Nevertheless, overall, patients did not view the process of discontinuing these drugs as a significant event during their GDH admission.”* Ambulatory setting *[12]* |
|  | GPs’ frustration | *“it is an effort and time and frustration trying to get people off of these things”* Interview of a GP, ambulatory setting [5] |
| **Legend: BZRA**: Benzodiazepine receptor agonists, **GP:** General Practitioner, **TDF**: Theoretical Domains Framework | | |

- - 1. Anthierens, S.; Grypdonck, M.; De Pauw, L.; Christiaens, T. Perceptions of nurses in nursing homes on the usage of benzodiazepines. *Journal of clinical nursing* **2009**, *18*, 3098-3106.
  - 2. Bourgeois, J.; Elseviers, M.M.; Azermai, M.; Van Bortel, L.; Petrovic, M.; Vander Stichele, R.R. Barriers to discontinuation of chronic benzodiazepine use in nursing home residents: Perceptions of general practitioners and nurses. *Eur. Geriatr. Med.* **2014**, *5*, 181-187.
  - 3. Williams, F.; Mahfouz, C.; Bonney, A.; Pearson, R.; Seidel, B.; Dijkmans-Hadley, B.; Ivers, R. A circle of silence: The attitudes of patients older than 65 years of age to ceasing long-term sleeping tablets. *Australian Family Physician* **2016**, *45*, 506-511.
  - 4. Lasserre, A.; Younès, N.; Blanchon, T.; Cantegreil-Kallen, I.; Passerieux, C.; Thomas, G.; Chan-Chee, C.; Hanslik, T. Psychotropic drug use among older people in general practice: discrepancies between opinion and practice. *The British journal of general practice : the journal of the Royal College of General Practitioners* **2010**, *60*, e156-162.
  - 5. Cook, J.; Marshall, R.; Masci, C.; Coyne, J. *Physicians' perspectives on prescribing benzodiazepines for older adults: a qualitative study*; 2007; Volume 22, pp. 303-307.
  - 6. Martin, P.; Tannenbaum, C. A realist evaluation of patients' decisions to deprescribe in the EMPOWER trial. *BMJ Open* **2017**, *7*.
  - 7. Kuntz, J.; Kouch, L.; Christian, D.; Peterson, P.; Gruss, I. Barriers and Facilitators to the Deprescribing of Nonbenzodiazepine Sedative Medications Among Older Adults. *The Permanente journal* **2018**, *22*, 17-157.
  - 8. S, I.; HV, C.; R, C.; SC, Y.K.; S, F.; B, W. Attitudes to long-term use of benzodiazepine hypnotics by older people in general practice: findings from interviews with service users and providers. *Aging & mental health* **2004**, *8*, 242-248.
  - 9. Barter, G.; Cormack, M. The long-term use of benzodiazepines: patients' views, accounts and experiences. *Family practice* **1996**, *13*, 491-497.
  - 10. Cook, J.; Biyanova, T.; Masci, C.; Coyne, J. Older patient perspectives on long-term anxiolytic benzodiazepine use and discontinuation: a qualitative study. *Journal of general internal medicine* **2007**, *22*, 1094-1100.
  - 11. Lambson, M.A. Benzodiazepine use in elderly residents od a retirement village in Sandton: knowledge, attitudes and perceptions of the patients, the nursing staff and the prescribing doctors University of the Witwatersrand, Johanesbourg, 2003.
  - 12. Chen, L.; Farrell, B.; Ward, N.; Russell, G.; Eisener-Parsche, P.; Dore, N. Discontinuing benzodiazepine therapy: An interdisciplinary approach at a geriatric day hospital. *Can. Pharm. J.* **2010**, *143*, 286-295.
